# A meta-core outcome set for stillbirth prevention and bereavement care following stillbirth in LMIC

**DOI:** 10.1136/bmjgh-2024-017688

**Published:** 2025-01-28

**Authors:** Kushupika Dube, Farai Marenga, Elizabeth Ombeva Ayebare, Carol Bedwell, Nasim Chaudhry, Idesi Chilinda, Angela Chimwaza, Declan Devane, Sudhindrashayana Fattepur, Unice Goshomi, Tayyeba Kiran, Rose Laisser, Tina Lavender, Tracey A Mills, Allen Nabisere, Zaib Un Nisa, Bellington Vwalika, Sabina Wakasiaka, Jamie J Kirkham

**Affiliations:** 1 Mpilo School of Midwifery, Mpilo Central Hospital, Bulawayo, Zimbabwe; 2 Women’s University in Africa, Harare, Zimbabwe; 3 Department of Nursing, Makerere University, Kampala, Uganda; 4 Department of International Public Health, Liverpool School of Tropical Medicine, Liverpool, UK; 5 Pakistan Institute of Living and Learning (PILL), Karachi, Pakistan; 6 Kamuzu University of Health Sciences, Lilongwe, Malawi; 7 School of Nursing and Midwifery, University of Galway, Galway, Ireland; 8 HRB-Trials Methodology Research Network, University of Galway, Galway, Ireland; 9 Department of Paediatrics, Karnataka Institute of Medical Sciences, Karnataka, India; 10 Archbishop Anthony Mayala School of Nursing, Catholic University of Health and Allied Sciences, Mwanza, Tanzania; 11 School of Medicine, University of Zambia, Lusaka, Zambia; 12 School of Nursing Sciences, University of Nairobi, Nairobi, Kenya; 13 Centre for Biostatistics, The University of Manchester, Manchester Academic Health Science Centre, Manchester, UK

**Keywords:** Maternal health, Obstetrics

## Abstract

**Study objective:**

Stillbirth is burdensome in low-income and middle-income countries (LMICs), especially in sub-Saharan Africa and South Asia. Currently, there are two core outcome sets (COS) for stillbirth (prevention and bereavement care), but these were developed with limited reflection of the needs of parents in an LMIC setting. To address this gap, the objective of this study was to establish consensus on the most important outcomes for stillbirth prevention and bereavement care following stillbirth in sub-Saharan Africa and South Asia.

**Methods:**

Previous stillbirth outcomes were reviewed for inclusion into the COS by senior research leaders and community engagement and involvement members from six sub-Saharan African and two South Asian countries. An online real-time Delphi survey was then conducted with healthcare professionals, parents who have experienced a stillbirth and researchers in the field to score the agreed list. The results of the Delphi were summarised and then discussed at a virtual consensus meeting where the final COS were agreed.

**Results:**

287 participants contributed towards the Delphi (143 midwives, 32 obstetricians, 50 mothers, 12 fathers and 50 researchers), with at least 2 parents attending the full consensus meetings. Consensus was reached on 13 core outcomes for stillbirth prevention covering 5 domains: obstetric, fetal, perinatal and neonatal outcomes and maternal complications. For bereavement care following a stillbirth, five core outcomes reached a consensus, which included outcomes related to labour and birth, a postpartum complication, care experience, mental health and emotional and social well-being.

**Discussion:**

These COS will improve the consistency of outcomes for future research in an LMIC setting. Additionally, they will complement existing COS for stillbirth prevention and bereavement care developed in high-income settings. The output from this work will move us towards a global set of outcomes that can be used in stillbirth research worldwide.

**WHAT IS ALREADY KNOWN ON THIS TOPIC**

- Core outcome sets (COS) for stillbirth prevention and bereavement care following stillbirth have been developed previously but they did not involve meaningful contributions from low-income and middle-income countries (LMICs). Given the vast majority of stillbirths occur in LMIC settings and because there are significant resource and societal differences between LMIC and high-income countries (HICs), a COS for the study of stillbirth specific to LMIC is needed.

**WHAT THIS STUDY ADDS**

- 13 outcomes have been included in the COS for stillbirth prevention across 5 domains, which reflect outcomes relating to childbirth, the fetus, the perinatal and neonatal periods and maternal-related complications. Five outcomes were included in the COS for bereavement care and related to the labour and birth, an important complication shortly after birth (obstetric fistula), the care experience, mental and emotional status of the parents and social outcomes.
- The type of stillbirth (antepartum or intrapartum) featured in both the prevention and bereavement care COS. Six core outcomes for prevention and two for bereavement care were common to those COS developed with predominantly a HIC setting in mind and this LMIC study.

**HOW THIS STUDY MIGHT AFFECT RESEARCH, PRACTICE OR POLICY**

- These stillbirth prevention and bereavement care outcomes will inform future research and help improve both the understanding of preventative strategies and the provision of high-quality bereavement care after stillbirth in an LMIC setting by helping promote consistency in outcome selection and reporting.
- The overlapping core outcomes identified in this, and previous studies will help inform outcome choice for global research studies in stillbirth spanning both HIC and LMIC.

## Introduction

 In 2021, it was reported that an estimated 1.9 million stillbirths occurred annually with most occurring in sub-Saharan Africa and South Asia.^1^ Determining the cause of stillbirth can be challenging, though in a low-income and/or middle-income country (LMIC) setting, it is often associated with prolonged and obstructed labour, maternal complications and abnormal presentations.^2^ Global targets for stillbirth reduction are set out by The Every Newborn Action Plan of 2014, where it is hoped that all countries achieve stillbirth rates of 12 or fewer per 1000 total births by 2030.^3^ To achieve this, the WHO is committed to increasing the understanding of preventative stillbirth strategies, reducing the stigma surrounding stillbirth as well as strengthening health systems to provide high-quality antenatal, intrapartum and bereavement care. Accurate estimates of the burden, as well as the development, implementation and evaluation of clinical, diagnostic, therapeutic and care-related interventions, are required to support the evidence base for improving clinical practice to reduce preventable deaths and optimise experiences.^4^

Implementing core outcome sets (COS), a minimum set of outcomes that should be measured and reported in all clinical trials undertaken in a specific health condition would help improve the quality of this evidence base.^5^ Two COS for stillbirth already exist: one for stillbirth prevention (COSTIL COS)^6^ and one for bereavement care following stillbirth (iCHOOSE).^7 8^ These two studies are described in more detail in the subsequent section, but their development had little involvement with participants from within an LMIC setting, especially from parents. The transferability of COS developed in a predominantly high-income setting for use in an LMIC is largely unknown. Consequently, there is uncertainty about whether these COS should be adopted in a different setting due to contextualisation, resource and societal differences.

The overall aim of this work is to determine core outcomes for stillbirth prevention and perinatal bereavement care (capturing parents who have experienced the death of a baby before, during and soon after birth) for use in sub-Saharan Africa and South Asia and to assess the relevance of existing COS in these settings. Specific objectives are to:

1. Identify suitable outcomes that have relevance in stillbirth care in sub-Saharan Africa and South Asia through review and discussion of outcomes included in previously related COS.
2. Assess the importance of these identified outcomes among key stakeholders (parents, obstetricians, nurse midwives and researchers) in six sub-Saharan African countries and two countries in South Asia using a real-time Delphi (RTD) technique.
3. Conduct a consensus-building process to determine which outcomes should be included in the final COS for interventions and care options for stillbirth prevention and bereavement care following stillbirth for use in LMIC settings.
4. Develop recommendations on key stillbirth outcomes that could be useful to measure in both an LMIC and high-income country (HIC) settings.

### Stillbirth COS

Two COS for stillbirth have been developed: one for stillbirth prevention (COSTIL) in 2021^6^ and one for bereavement care after stillbirth (iCHOOSE) developed in 2022.^8^ Both studies used systematic review and interview methods to generate their potential list of outcomes, ensuring both healthcare professionals’ and patients’ views were considered. Delphi surveys and consensus meetings were used in both for reaching consensus on their respective final COS.

For the COSTIL COS, 96% (124/129) of the participants (clinicians (n=70), researchers (n=34) and parents (n=25)) responding to the first round of the Delphi survey were based in HICs, with just five having experience in practice and research from an upper-middle or low-income setting. No parents from an LMIC region were included in this COS development. 11 outcomes were included in the final COS with five relating to the mother: (1) fetal loss, (2) onset of and mode of delivery, (3) maternal mortality or near miss, (4) psychological and social impact on the women and (5) women’s knowledge and six outcomes relating to the baby: (1) timing of stillbirth, (2) neonatal mortality, (3) gestational age at delivery, (4) birth weight, (5) congenital anomaly and (6) neonatal intensive care unit/special care baby unit or other higher-level neonatal care length of stay.

For the iCHOOSE COS, 381 parents or family members and 192 professionals (including researchers) engaged in the first round of the Delphi, with just 5 parents/family members and 17 professionals representing LMIC participating. However, only 64% (14/22) of those from these LMIC settings went on to complete all rounds of the Delphi survey. LMIC participation at the consensus meetings was low, with just four professionals (three from Ghana and one from India) and no parent/family member representation. Eight outcomes were included in the final core set as mandatory outcomes in all circumstances: (1) life-threatening complications and death, (2) parents’ experience of respectful and supportive care, (3) grief, (4) mental health and emotional well-being, (5) isolation, (6) stigma, (7) impact on work and (8) impact on the relationship with immediate family.

## Methods

### Study registration and study oversight

The study was prospectively registered with the Core Outcome Measures in Effectiveness Trials Initiative^9^ and is reported in line with the COS-STAR(Core Outcome Set–STAndards for Reporting) reporting guidance.^10^ The study steering committee (SSC) comprised membership from a multidisciplinary team within a National Institute for Health and Care Research (NIHR) Global Health Research Unit (GHRU) on the Prevention and Management of Stillbirths and Neonatal Deaths in sub-Saharan Africa and South Asia. This international committee included healthcare professionals (within-country leads), within-country community engagement and involvement (CEI) representatives, including bereaved parents (mothers and fathers) and methodologists (inclusive of a COS development expert) representing six sub-Saharan African countries (Kenya, Malawi, Tanzania, Uganda, Zambia and Zimbabwe), two countries in South Asia (Pakistan and India) and UK experts with substantial collaborative research experience in an LMIC setting. Members of the COSTIL and iCHOOSE COS were also added to the SSC.

### COS development methods

The development of the two COS involved three stages: stage 1: identification of the long list of outcomes; stage 2: outcome prioritisation and stage 3: a consensus meeting and final COS selection. A study protocol for this study is available in Dube *et al*^11^ and, therefore, we describe the methods to each stage in brief below alongside any protocol deviations.

### Stage 1: outcome list generation

To avoid duplication of effort and because the original studies followed the minimum standards for COS development,^12^ the list of outcomes and outcome domains used in this study were repurposed from the COSTIL study for stillbirth prevention (58 outcomes) and the iCHOOSE study for bereavement care following stillbirth (108 outcomes). To enhance this list and to ensure the appropriateness of the list in accordance with an LMIC setting, a series of ‘think-aloud’ interviews were carried out with country leads and CEI members from each participating country. The think-aloud approach has been used successfully for COS development studies for stillbirth previously to plan the Delphi questionnaire,^8^ but here we used the approach to aid the reduction and refinement of outcomes contained within the long list. Participants were presented with the outcome lists, plain language summaries (where available) and final Delphi results from the original studies, and for each outcome, asked the question, ‘Based on the information provided, how relevant/feasible do you think it is to measure each outcome for all stillbirth occurrences in an LMIC setting?’ During the interviews, participants could also make suggestive changes to the outcomes descriptors used or suggest new outcomes not currently listed. The final outcome list and plain language summaries (outcome definitions) was agreed on following a meeting with all country leads and CEI members in attendance, such that the outcomes could be widely understood by all stakeholders groups in stage 2. During this meeting, we also discussed the level of granularity of the outcome domains. We considered options for merging and dropping outcomes to ensure it was practically feasible for participants to score all outcomes in the Delphi survey in stage 2.^13^

### Stage 2: outcome prioritisation

#### Stakeholders

Stakeholder groups representing healthcare professionals (eg, obstetricians and nurse midwives), researchers and parents were invited to complete an RTD^14^ survey to score each outcome using Calibrum (Surveylet) software.^15^ Participants were invited through the NIHR GHRU network, previous NIHR Group and through country lead contacts. Each participant identified contributed to both the COS for stillbirth prevention and bereavement care following stillbirth.

#### Consensus process (RTD survey)

The RTD remained open for 8 months to allow for sufficient recruitment and access to internet facilities. It also provided additional within-country support from country leads and their researchers to help participants complete the survey when needed. Using suitable demarcation, the listed bereavement care outcomes followed directly after the prevention outcomes. Participants were asked to rate outcomes based on their importance for inclusion in the COS, using a 9-point Likert scale, with one meaning not important and nine meaning critical for inclusion in the COS. Participants were also given the option to select ‘I don’t know’ if they felt they could not score a particular outcome. On scoring each outcome, participants were presented with graphs showing how the outcomes were rated overall and by each stakeholder group immediately after they had rated the outcome for the first time. There were no limits on how many times participants could revisit the survey, see updates on how outcomes were rated and rescore outcomes if they chose to do so. Participants were emailed to remind them that they could revisit the survey periodically and asked to score any additional outcomes that were added for the first time which were included by agreement of the SCC.

### Stage 3: consensus meeting

The results of the Delphi survey were summarised according to a predefined consensus criteria outlined in table 1. Two changes were made to the consensus meeting process from the original protocol. First, for logistical reasons and because the meeting timings did not align with organised face-to-face meetings with the unit, the consensus meetings were held online on Zoom. Second, as many outcomes reached the ‘consensus in’ criteria following the Delphi survey, the advice of the International Advisory Meeting for the unit to help facilitate the meeting was to invite consensus meeting participants to rank their top 10 outcomes in terms of critical importance for each COS as part of a pre-meeting exercise. This exercise was done in line with James Lind Alliance priority setting principles.^16^ All outcomes were taken forward and considered at the consensus meeting.

**Table 1:** Consensus criteria

Consensus classification	Description	Definition
Consensus in	Consensus that the outcome should be included in the final core outcome set.	70% or more participants scoring as 7–9 (critical) and <15% participants scoring as 1–3 (limited importance) in all stakeholder groups.
Consensus out	Consensus that the outcome should not be included in the final core outcome set.	50% or fewer participants scoring 7–9 (critical) in all stakeholder groups.
Equivocal	Uncertainty about the importance of the outcome.	All other responses.

SSC members and selected participant members representing similar numbers from all stakeholder groups from LMIC across the NIHR GHRU Network were invited to the consensus meeting which was held on separate days for bereavement care (25 April 2024) and stillbirth prevention (26 April 2024). An experienced COS developer with experience in pregnancy and childbirth chaired the meeting. During the meeting, summaries of the final Delphi results were provided alongside a summary of the ranking exercise. Participants were invited to briefly discuss the importance of each outcome if they wished before voting anonymously on including the outcome in the final COS. Participants were asked to vote as either ‘not important’ (aligned to 1–3), ‘important’ (aligned to 4–6) or ‘critical’ (aligned to 7–9) on the Delphi scale. For the outcome to be preliminary included in the final core set, 80% or more of the participants needed to vote the outcome as critical. The increase in the threshold from the Delphi stage was described in the protocol^11^ and was aimed at facilitating a smaller number of core outcomes in the final set which users of the COS are more likely to measure.

#### Final COS

At the end of the consensus meeting, the panel reviewed the proposed outcomes voted to be included in the COS. A final reflective discussion was held to ensure the included outcomes were pragmatic and feasible to measure in an LMIC setting as well as agreeing on the outcome definitions. The discussion further considered whether there was a good balance of outcomes in the core sets, which reflected outcomes related to the mother, offspring and the core areas of healthcare indicated by the outcome domains.

#### Meta-COS

The final recommended outcomes for inclusion in the final COS from this study were compared with those from COSTIL and iCHOOSE. Complementary outcomes identified as core across these independent studies will be considered as meta-core outcomes for use in stillbirth research and clinical practice (prevention and bereavement care) in both high-income and LMIC settings.

### Patient and public involvement

CEI members including bereaved parents (mothers and fathers) from each participating country formed part of the steering committee overseeing the study. As well as participating in the study, CEI members were involved in the design and recruitment of other parent participants into the study. Parent participation was included in each of the three stages of the COS development process.

## Results

The final COS for stillbirth prevention included 13 outcomes across 5 outcome domains and 5 outcomes from 5 domains for bereavement care following stillbirth (table 2).

**Table 2:** Outcomes included in the stillbirth prevention and bereavement care following stillbirth core outcome set for use in LMIC

Stillbirth prevention		
Domain	Outcome	Description
Obstetric outcome	Mode of birth	How did the woman give birth? (eg, caesarean section, spontaneous vaginal, assisted vaginal birth (forceps or ventouse).
	Pre-eclampsia	Multisystem progressive disorder characterised by new onset hypertension and proteinuria diagnosed after 20 weeks gestation by clinical assessment/signs and/or laboratory results.
	Type of stillbirth	Did the death of the fetus occur in the during pregnancy and before labour (antepartum) or during labour and before birth (intrapartum)?
Fetal outcome	Reduced fetal movements	Maternal perception of reduction/cessation of fetal movements (self-report).
	Emergency birth for fetal compromise	Fetus requiring immediate birth due to clinical signs of compromise (eg, abnormal foetal heart rate pattern).
Perinatal outcome	Stillbirth	A fetus that dies in utero after 28 weeks of pregnancy (before or during birth).
	Birth weight	The weight of the baby immediately following birth (measured in grams).
Maternal complication	Eclampsia	Onset of seizures in a women with pre-eclampsia (clinical observation).
	Placental abruption	Premature separation of the placenta before the fetus is born (clinical observation).
	Antepartum haemorrhage	Bleeding from the genital tract occurring from 28 weeks of pregnancy and prior to the birth of the baby (clinical observation).
	Severe maternal infection	Invasion of maternal tissue by pathogens and systemic host response requiring hospital admission (identified by clinical assessment/laboratory results).
Neonatal outcome	Gestational age at birth	Measure of the age of a pregnancy from the woman's last menstrual period or other means (eg, via ultrasound).
	Neonatal mortality	Death of a baby within first 28 days of life.

Bereavement care following Stillbirth		
Domain	Outcome	Description
Labour and birth outcome	Type of stillbirth	Did the death of the fetus occur during pregnancy and before labour (antepartum) or during labour and before birth (intrapartum)?
Postpartum medical outcome	Obstetric fistula	An abnormal connection between a woman’s genital tract or rectum directly linked to obstructed labour (diagnosed by clinical assessment).
Care experience outcome	Parents' experience of care and support	Determined qualitatively between researcher and mother/father or via exit interviews or post-discharge survey to reflect both positive and negative experiences.
Mental health and emotional outcome	Mental health and emotional well-being	Measurement of depressive symptoms, anxiety symptoms, mental health difficulties.
Social outcomes	Social well-being	Measurement of interpersonal relationships (sense of belonging, social inclusion and stability).

LMIClow-income and middle-income country

### Deciding on the long list of outcomes

A summary of the outcome list generation stage is provided in online supplemental file 1 (prevention) and online supplemental file 2 (bereavement). For prevention, 20 outcomes were removed from the original list used in the COSTIL study because they were either rated as having very little importance in this previous study, were not considered relevant to an LMIC setting, were not supported by the ‘think-aloud’ interviews or were deemed linked to other outcomes already included. ‘Stigma’ surrounding stillbirth was the only new outcome added, resulting in 39 prevention outcomes for the RTD.

For bereavement care, all outcomes from the following three domains in the iCHOOSE study were excluded: ‘twin or multiple outcome’, ‘subsequent pregnancies’ and ’subsequent children’ because none of these outcomes were included in the iCHOOSE COS and were deemed out of scope and addressed a different research question. Several other outcomes were excluded for reasons similar to those for stillbirth prevention (eg, not relevant in an LMIC setting). It was also agreed to combine several outcomes because they were deemed too granular for inclusion. For example, in iCHOOSE, there were several outcomes related to ‘grief’, how this was managed and how different family members experienced this. In this study, these were grouped as a single outcome, ‘grief’. No new outcomes were introduced, resulting in 22 bereavement outcomes to include in the RTD.

### Online RTD process

287 participants contributed to the RTD survey with 174 (61%) scoring all outcomes and 230 (80%) completing at least half of the survey. The median number of visits to the survey was 2; IQR (1–3). Participant demographics are presented in table 3 and comprised 61% healthcare professionals, 22% parents and 17% researchers. While there appeared to be over-representation of participants from Uganda (and in particular from nurse midwives), the median percentage of scores achieving the consensus criteria across all the outcomes for prevention and bereavement care was within the range of those for all other countries. At the end of the Delphi process, the newly added ‘Stigma’ outcome did not reach the consensus criteria for any stakeholder group for inclusion in the stillbirth prevention COS. For prevention, a further three outcomes (postpartum haemorrhage, antenatal/postnatal depression and social isolation) did not meet the criteria for both the nurse midwives and researcher group. In comparison, three further perinatal outcomes, all four neonatal complications, two neonatal outcomes and one health service outcome also did not meet the criteria for inclusion as rated by researchers (online supplemental file 3). All 21 outcomes met the predefined criteria for inclusion in the stillbirth bereavement care COS across all three stakeholder groups (online supplemental file 4). The Delphi participants made no new outcome suggestions.

**Table 3:** Participant Characteristics for the Real-Time Delphi

	Healthcare professionals		Parents		Researchers
Country	Obstetrician	Nurse Midwife	Mother	Father	
Africa					
Kenya	1	5	3	0	4
Malawi	1	8	12	0	4
Tanzania	1	9	4	1	3
Uganda	10	97	13	4	10
Zambia	1	0	0	0	2
Zimbabwe	5	19	7	1	7
South Asia					
India	9	2	5	0	6
Pakistan	3	3	6	6	10
UK	1	0	N/A	N/A	4
Total n(%)	32 (11%)	143 (50%)	50 (17%)	12 (4%)	50 (17%)

### Consensus meeting

All countries and stakeholder groups were represented in the ranking exercises or the consensus meetings. The ranking results that fed into the consensus meeting discussion are shown in online supplemental file 5 and online supplemental file 6 for stillbirth prevention and bereavement care following stillbirth, respectively. 14 voting participants attended the consensus meeting on stillbirth prevention (6 midwives, 1 obstetrician, 2 CEI/parent members and 5 researchers). A summary of the voting for the consensus meeting and important discussion points is presented in online supplemental file 7. In addition to wording changes, ‘hypertension’ was replaced by ‘pre-eclampsia’, agreed as a more specifically defined disorder that was more likely to be associated with stillbirth. The outcome ‘chorioamnionitis’ was considered important but refined to capture a broader range of infection-related complications labelled as ‘severe maternal infection’.

15 voting participants attended the consensus meeting on bereavement care following stillbirth (7 midwives, 2 obstetrician, 3 CEI/parent members and 3 researchers). A summary of the voting and key decisions can be found in online supplemental file 8. Similarly to prevention, there were some wording changes. The broader outcome of ‘complications during the birth for the mother or baby’ was changed to the specific complication of ‘obstetric fistula’. Neonatal complications did not reach a consensus for inclusion in the final COS. Physical injury to the baby was also discussed as an important complication but this did not reach consensus for inclusion in the final COS. None of the individual social outcomes reached the consensus criteria but on final reflection, it was felt that something around social care was missing from the final COS. It was felt that the social outcomes considered were too specific and not well defined, and a new outcome of ‘social well-being’ was proposed and agreed to be critical to include in the final COS.

### Meta-COS

**Figure 1:**
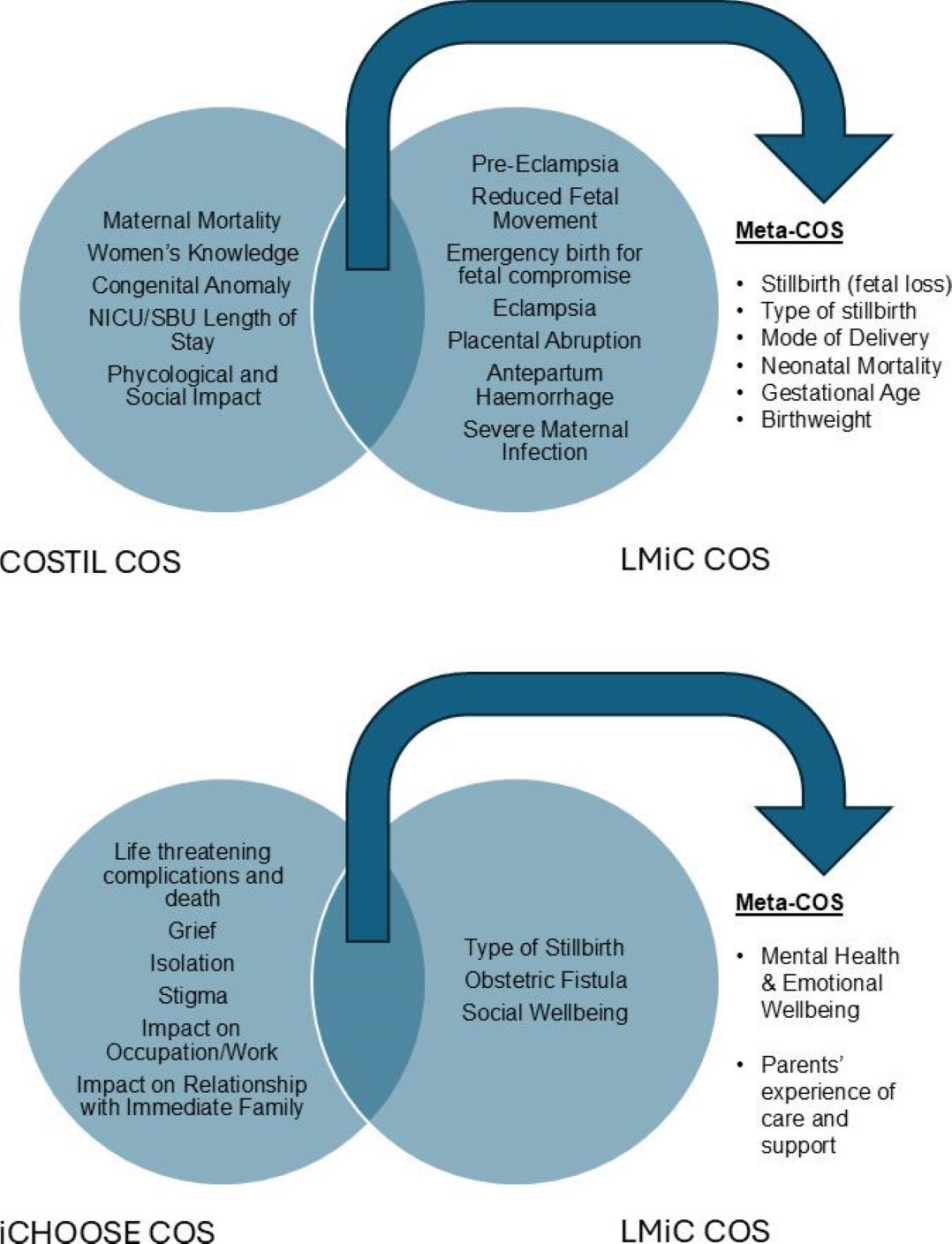
Meta-core outcome set (COS) for stillbirth prevention and bereavement care following stillbirth. LMIC, low-income and middle-income country; NICU, neonatal intensive care unit; SBU, special care baby unit.

Figure 1 shows that there are six overlapping outcomes for stillbirth prevention (upper panel) and two outcomes for bereavement care (lower panel).

## Discussion

This study has developed a healthcare professional, researcher and parent consensus on outcomes for use in research and routine data collection for stillbirth prevention and bereavement care following stillbirth in an LMIC setting. Through the methods used, the exclusivity of including mostly LMIC participants in this study and extensive discussion, we can be assured that the chosen core outcomes are both applicable and are of practical use in an LMIC setting. Our study also demonstrates that several core outcomes prioritised in an HIC setting are transferable to LMIC, while others may be less appropriate (figure 1). We recommend that future studies aiming to prevent or evaluate care after stillbirth use the core outcomes relevant to the setting as a minimum while carefully considering other outcomes that may be useful. Where core outcomes overlap from COSTIL and iCHOOSE, the ‘meta-COS’ may be a helpful starting point for outcome selection for studies conducting research in both an HIC and LMIC setting. The overlap in outcomes between studies is encouraging and confirms these outcomes have been deemed critical for inclusion in a COS from independent consensus processes containing different participants. While there is optimism that the meta-COS is a positive step in moving towards a global COS, we note caution that there are some significant areas of the world that were not meaningfully included in either of these COS development studies, for example, South America.

The uptake of COS varies widely across health conditions.^17^ One of the common barriers to uptake often relates to the lack of consensus on the instruments that should be used to assess each core outcome. Using formal assessment methods^18^ for selecting appropriate instruments for measuring each core outcome was beyond the scope of this current study because specifying options for ‘how’ core outcomes should be measured is seen as a separate second step in the COS development process. However, the study group was able to make some preliminary informal recommendations on outcome measurement instruments (OMIs) for the core outcomes based on current knowledge. For the stillbirth prevention outcomes, this was relatively straightforward as all outcomes would require only the recording of a specific event, which would often be determined by clinical assessment/signs/observation/scan and, in some cases, laboratory results. Birth weight would be measured using appropriate measuring scales and reported in grams. For bereavement care, the type of stillbirth is common to the prevention COS, obstetric fistula would again be measured using clinical assessment while the parents’ experience of care outcome would typically be measured as an exit interview (for research) or a postdischarge survey in the case of routine practice. Both the mental health and emotional well-being and social well-being outcomes would require some form of valid measurement instrument to capture these outcomes. At this time, and without any formal evaluation, we cannot confidently recommend any specific tool for use in measuring the mental health/emotional well-being and social well-being outcomes, respectively. Previous studies have suggested numerous tools in use for evaluating mental health and psychosocial well-being^19^ although many are not specific to an LMIC setting and/or stillbirth. Subsequently, many of these tools show promise but would require validation for cultural diversity and context within both an LMIC and childbirth clinical setting.^20^ In the interim, we have identified a social well-being scale that has been validated in South Africa,^21^ as well as other instruments (Patient Health Questionnaire-9 (PHQ-9)),^22^ Generalised Anxiety Disorder scale- (GAD-7),^23^ Perinatal Grief Intensity scale,^24^ Edinburgh postnatal depression scale^25^ that are commonly used across the globe and could be considered to measure these core outcome domains.

Strengths of this COS study include the use of methods meeting the COS-STAD^12^ recommendations, preprinting the study protocol before undertaking the study^11^ as well as adopting RTD methodology to help reach consensus on the final core outcomes. This approach was particularly advantageous in this setting given the challenges of capturing participants in the community on more than one occasion, which is typically required using traditional round-based Delphi methods. This study also engaged with nearly 300 participants representing multiple stakeholders across eight separate LMIC, including parents, and we were able to overcome many of the challenges cited for low initiation of LMIC stakeholders within the COS development process.^26 27^ Despite this success, the study was limited to include only LMIC in Sub-Saharan Africa and South Asia which were allied partners within our GHRU. While there are many other countries in sub-Saharan Africa and South Asia that were not considered, there is no reason to believe that opinion would differ in these similar settings.

This study provides key learning for those looking to engage LMIC stakeholders and sustain participation within their COS development studies. These experiences and ‘tips’ will be subject to a separate publication. In brief, the success of this study was based mainly on long-standing established research relationships between the UK researchers and LMIC partners. One limitation of the study is that parent participation in the final consensus meeting was low, with the small risk that the consensus and final COS might not reflect their reviews. However, our approach ensured that their views were included in all consensus-building stages, which included extensive feedback from CEI members in the meeting following the ‘think-aloud’ interviews. The selection of outcomes for the COS in the final reflective discussion following the consensus meeting also took into account their importance for all stakeholder groups, considering the perspectives of those who had experienced a stillbirth. The final agreed COS was also circulated to all Delphi participants for comment, acceptance of what was agreed and for dissemination purposes. One further shortcoming is that the COS development process was conducted in the English language only, which may not have been the first-choice language for many of our participants. Through our CEI work, we were confident that most participants were proficient enough in the English language for our purposes within our geographical setting, and we offered within-country support from country leads to help participants with language barriers when this was needed. Language translations in COS development studies remain problematic with the extra effort required often not translating in terms of meaningful numbers of additional participants.^28^

Further work stems from the above discussion as there is a possible need to examine how applicable this meta-COS is in areas of the world that have not been considered in the COS development process. This could involve further work with stakeholders in different geographical locations on how the COS may need to be further developed to specify any variations to make it more widely applicable, including the adding or removing of any outcomes for these other settings. While informal recommendations were made in this study about the specification of OMIs for each outcome, there is a need for future work to set out valid and reliable OMIs for each outcome where it is needed, with the knowledge that few existing available tools are likely to have been validated in an LMIC setting. A tool for perinatal bereavement care for use in LMIC is currently under development^29^ and on completion should form part of the evaluation as to whether this is an optimal OMI for this current recommended COS.

## Conclusion

This study has developed two COS specifically for LMIC to include in all intervention studies focusing on stillbirth prevention and aftercare following stillbirth. These COS comprise relevant outcomes that have been carefully selected using recommended standards and, therefore, are likely to be relevant and meaningful to a wide range of LMIC stakeholders, including parents. Combined with similar COS developed for a predominantly high-income setting, use of the meta-COS will guide and inform routine clinical practice and research by providing the opportunity to promote consistency in outcome selection. Subsequently, this will lead to the emergence of higher levels of evidence to ensure the comparability of effectiveness across studies investigating preventative stillbirth strategies, and improving healthcare systems to provide high-quality bereavement care internationally. Future work must focus on seeking consensus on how the core outcomes should be measured, especially for the bereavement care outcomes, and to provide guidance on this for researchers.

## supplementary material

10.1136/bmjgh-2024-017688 online supplemental file 1

10.1136/bmjgh-2024-017688 online supplemental file 2

10.1136/bmjgh-2024-017688 online supplemental file 3

10.1136/bmjgh-2024-017688 online supplemental file 4

10.1136/bmjgh-2024-017688 online supplemental file 5

10.1136/bmjgh-2024-017688 online supplemental file 6

10.1136/bmjgh-2024-017688 online supplemental file 7

10.1136/bmjgh-2024-017688 online supplemental file 8

## Data Availability

All data relevant to the study are included in the article or uploaded as online supplementary information.
