# Supplementary material for: A meta-core outcome set for stillbirth prevention and bereavement care following stillbirth in LMIC
Source: BMJ Glob Health. 2025 Jan 28;10(1):e017688. doi: 10.1136/bmjgh-2024-017688 (PMC11781104; doi:10.1136/bmjgh-2024-017688)
Supplement: online supplemental file 1 [file bmjgh-10-1-s001.pdf]

Supplementary Table 1a: Outcomes and descriptors to include in the real-time Delphi survey: stillbirth prevention.

		Clinicians (from COSTIL)		Parents (from COSTIL)		Researchers (from COSTIL)		Think Aloud/Meeting Discussion	Comments
**Outcomes used in COSTIL COS**	**Outcome descriptors (co-produced by study team (including CEI members)**	**Count**	**Est. % (7to 9)**	**Count**	**Est. % (7to 9)**	**Count**	**Est. % (7to 9)**	**Include in new Delphi ?**	
**Obstetric Outcome**									
Spontaneous preterm birth	This is when a woman gives birth earlier than the expected due date (could be one or two months)	52	87%	11	66%	26	88%	Yes	Include
Mode of delivery	The method the woman gave birth e.g. vaginal, caesarean section, etc.	52	41%	11	44%	26	42%	Yes	Include
Induction of labour	A woman is given drugs to go into labour e.g. to save the baby or the woman	52	35%	11	37%	26	35%	No	Remove: Limited importance in COSTIL study. Not always relevant
Mother-infant interaction	A communication or bond between the mother and baby	52	23%	11	57%	26	20%	No	Remove: Limited importance in COSTIL study
Timing of stillbirth (antepartum or intrapartum)	Describes the period when the baby was born lifeless, pregnancy or birth	Not available		Not available		Not available		Yes	Include: Added at the consensus meeting in COSTIL study
Multiple gestational pregnancies	A pregnant woman who is carrying more than one baby in her womb	Not available		Not available		Not available		Yes	Include: Added in 2nd round of COSTIL Delphi study
Hypertension	This is when a woman's blood pressure is very high during pregnancy	52	58%	11	76%	26	51%	Yes	Include

**Fetal Outcome**									
Maternal reports of reduced fetal movements	This is when a pregnant woman reports reduced or excessive baby movements	52	52%	11	99%	26	59%	Yes	Include
Signs of compromise requiring emergency birth	A baby in the womb lacking oxygen and needs immediate birth	52	83%	11	92%	26	72%	Yes	Include
Biparietal Diameter	A measurement of a developing baby's head helps to estimate the baby's age	Not available		Not available		Not available		No	Remove: Added in 2nd round of COSTIL Delphi study.
Fetal movements in antenatal period	The movements made by the baby whilst in the womb	Not available		Not available		Not available		No	Remove: Added in 2nd round of COSTIL Delphi study. Not routinely monitored in LMIC
Harm to fetus from intervention	A baby in the womb is injured due to treatment given to the woman (e.g. external cephalic version, fundal pressure, unregulated induction of labour methods)	52	86%	11	92%	26	90%	Yes	Include
**Perinatal Outcome**									
Stillbirth	A baby born life less	52	98%	11	100%	26	100%	Yes	Include
Perinatal mortality	Death of a baby within seven days of life	52	97%	11	93%	26	99%	Yes	Include
Neonatal mortality	Death of a baby within one month of life	52	94%	11	85%	26	96%	Yes	Include
Birthweight	The weight measured immediately following birth of the baby	52	75%	11	58%	26	69%	Yes	Include
Miscarriage	Losing the pregnancy before the baby is able to survive on its own out of the womb	52	43%	11	62%	26	45%	Yes	Include. Supported by Think Aloud
Small for gestational age	A baby who is small compared to the months of pregnancy	Not available		Not available		Not available		Yes	New Outcome: Added after discussion at our meeting.
Gestational Diabetes Mellitus	A rise in the woman's blood sugar caused by pregnancy	Not available		Not available		Not available		Yes	Include: Added in 2nd round of COSTIL Delphi study
Gestational Age at Diagnosis of Stillbirth	The term describes the age of pregnancy when a baby was discovered to be lifeless	Not available		Not available		Not available		Yes	Include: Added in 2nd round of COSTIL Delphi study
Apgar <7 at 5 minutes	A test is done on the baby following birth to see normality	52	43%	11	57%	26	47%	Yes	Include: Supported by Think Aloud

**Maternal Complication**									
Preeclampsia	An abnormal rise in the blood pressure caused by pregnancy	52	74%	11	84%	26	63%	No	Remove: Linked to hypertension which is already included.
Eclampsia	Having a fit/convulsion during pregnancy due to high blood pressure	52	84%	11	84%	26	64%	Yes	Include
Maternal pulmonary oedema	A woman has a lot of fluid in the lungs causing breathing difficulty	52	62%	11	67%	26	31%	No	Remove: Limited importance in COSTIL study across all groups
Maternal renal impairment	A woman has poor kidney function	Not available		Not available		Not available		No	Remove: Not routinely monitored in LMIC
Haemolysis, elevated liver enzymes, low platelet count (HELLP)	A complication in pregnancy due to high blood pressure causing the breakdown of blood vessels and uncontrollable bleeding.	52	75%	11	76%	26	42%	No	Remove: Linked to hypertension which is already included.
Placental inefficieny	This is when the placenta is not functioning as expected to feed the baby and remove the waste products from the baby	Not available		Not available		Not available		No	Remove: Added in 2nd round of COSTIL Delphi study.
Placental abruption	Separation of a placenta before the baby is born can result from abdominal trauma, high BP, etc.	52	80%	11	84%	26	75%	Yes	Include
Chorioamnionitis	Infection of the placental membranes and the fluid inside them	52	74%	11	76%	26	42%	Yes	Include
Harm to mother from intervention	Manipulations causing harm to the mother	52	76%	11	60%	26	58%	Yes	Include: Supported by Think Aloud
Antepartum haemorrhage	A woman starts to bleed during advanced pregnancy before labour	52	52%	11	67%	26	47%	Yes	Include: Supported by Think Aloud
Postpartum haemorrhage	A woman bleeds a lot after birth of the baby	52	32%	11	59%	26	31%	Yes	Include: Supported by Think Aloud

**Maternal Outcome**									
Antenatal/postnatal depression	Feeling low or down during pregnancy or after giving birth	52	38%	11	63%	26	37%	Yes	Include: Supported by Think Aloud
Maternal mortality	Death of a mother as a result of pregnancy or childbirth	52	87%	11	64%	26	88%	Yes	Include
Social isolation	A woman is excluded from life experiences	52	45%	11	62%	26	46%	Yes	Include: Supported by Think Aloud
Childbirth experience	A woman's story involving events that happened during labour and birth	52	32%	11	52%	26	56%	Yes	Include: Supported by Think Aloud
Women's knowledge	What a woman knows in relation to pregnancy, labour and birth of her child	52	28%	11	87%	26	28%	Yes	Include: Supported by Think Aloud
Women's perceptions	A woman's ability to see, hear, or become aware of pregnancy, labour, and birth issues	52	32%	11	71%	26	36%	No	Remove: closely linked to women's knowledge
Women's attitudes	A woman's thinking or feeling towards pregnancy, labour and birth.	52	28%	11	69%	26	32%	No	Remove: closely linked to women's knowledge
Maternal: general health	This describes the well-being of the woman during pregnancy and after birth	52	35%	11	66%	26	38%	No	Remove: Limited importance in COSTIL study
Women's nutritional status	The state of a woman health during pregnancy, birth and post-partum	52	18%	11	52%	26	25%	Yes	Include: Supported by Think Aloud

**Neonatal Complication**									
Intraventricular haemorrhage	The baby is bleeding into the fluid-filled areas surrounded by the brain.	52	68%	11	61%	26	60%	Yes	Include: Supported by Think Aloud
Physical trauma	Examples of trauma include injury to the brain, limbs or bruises to the scalp causing bleeding	52	68%	11	70%	26	37%	No	Remove: not supported by Think Aloud
Early neurodevelopmental morbidity	An abnormal baby's brain development causes abnormal reflexes and functioning	52	67%	11	50%	26	53%	No	Remove: Limited importance in COSTIL study
Late neurodevelopmental morbidity	An abnormal baby's brain development causes noticeable delayed milestones	52	67%	11	64%	26	53%	No	Remove: Limited importance in COSTIL study
Gastrointestinal morbidity	A disease in a baby affecting the mouth, stomach, and anus can cause severe vomiting, diarrhoea, and death	52	44%	11	60%	26	25%	No	Remove: Limited importance in COSTIL study
Neonatal seizures	These are fits/convulsions in a baby due to infection, lack of oxygen to the brain etc.	52	70%	11	56%	26	53%	Yes	Include: Supported by Think Aloud
Respiratory distress syndrome	A baby fails to breathe after birth due to immaturity and the lungs still undeveloped	52	56%	11	59%	26	47%	Yes	Include: Supported by Think Aloud
Neonatal infection	Infection occurring in a baby due to an unclean cord, or infections acquired at birth	52	61%	11	61%	26	62%	Yes	Include: Supported by Think Aloud
Retinopathy of prematurity	An eye disease that can happen to a baby born premature affecting the eye sight	52	48%	11	60%	26	45%	No	Remove: Limited importance in COSTIL study

**Neonatal Outcome**									
Gestational age	The term describes weeks of pregnancy that the woman has.	52	87%	11	80%	26	91%	Yes	Include
Neonatal intensive care unit (NICU) admission	This is an emergency area where a newborn is admitted if they are unwell.	52	68%	11	82%	26	73%	Yes	Include
Congenital anomaly	These are defects that a baby is born with could be a large head, absence of body parts etc.	52	74%	11	73%	26	77%	Yes	Include
**Health Service**									
Health Service Costs	These are hospital bills incurred by the woman during pregnancy and after birth	52	35%	11	49%	26	21%	No	Remove: Limited importance in COSTIL study
Number of antenatal visits	The number of antenatal visits the mother had during pregnancy	52	36%	11	83%	26	46%	Yes	Include: important to parents in COSTIL study
Use of hospital resources (length of stay)	The cost of hospital resources used by the woman whilst in hospital after giving birth	52	35%	11	51%	26	31%	No	Remove: Limited importance in COSTIL study
Use of hospital resources (use of mechanical ventilation)	The cost of emergency resources used by the woman and her baby in assisting them to breathe	52	35%	11	69%	26	32%	No	Remove: Limited importance in COSTIL study
NICU/SCBU length of stay (days)	The number of days a newborn baby admitted to a high care centre	Not available		Not available		Not available		Yes	Include: supported over hospital resource (length of stay)
**Other**									
Stigma	Disgrace associated with giving birth to a lifeless baby	Not available		Not available		Not available		Yes	Include: New Outcome from Think Aloud which was preferred over 'psychological and social impact on mother' used in COSTIL
