# Supplementary material for: A meta-core outcome set for stillbirth prevention and bereavement care following stillbirth in LMIC
Source: BMJ Glob Health. 2025 Jan 28;10(1):e017688. doi: 10.1136/bmjgh-2024-017688 (PMC11781104; doi:10.1136/bmjgh-2024-017688)
Supplement: online supplemental file 2 [file bmjgh-10-1-s002.pdf]

Supplementary Table 1b: Outcomes and descriptors to include in the real-time Delphi survey: bereavement care following stillbirth.

Outcomes used in iCHOOSE COS	Outcome descriptors used in iCHOOSE COS (with amendments indicated)	Parents or family member (from iCHOOSE)		Professionals (from iCHOOSE)		Think Aloud/Meeting Discussion	
		Count	% (7to 9)	Count	% (7 to 9)	Include in new Delphi ?	Comments
**Labour and Birth Outcomes**							
Induction of birth	Was labour induced after the stillbirth was identified and did it work (if applicable)	35	26.50%	68	36.60%	No	Remove: Limited importance in iCHOOSE study. Not always relevant
~~Length of time from identification of a stillbirth to the birth~~ *New Outcome Type of stillbirth	~~How many hours or days it takes for the baby to be born after a stillbirth has been identified~~ Was the stillbirth identifed before the onset of labour (anterpartum) or after the onset of labour (intrapartum).	62	47.00%	123	61.50%	Yes	Change to 'Type of Still birth'. Original outcome cannot always be done as defined.
Pain relief for labour and birth	Was pain relief offered and used during labour and birth, for example, paracetamol, epidural	77	57.90%	129	65.50%	No	Remove: Limited importance in iCHOOSE study. Pain relief not always given.
Type of birth	For example, caesarean section, spontaneous vaginal, assisted vaginal birth (forceps or ventouse)	88	66.70%	131	65.80%	Yes	Include
Complications during birth for mother or baby	For example, long or obstructed labour, shoulders get stuck during birth, physical injury to mother such a third degree tear or physical injury to baby during birth	119	88.80%	163	83.20%	Yes	Include

**Postpartum medical outcomes**							
Maternal complications after birth	Complications experienced immediately after birth or following discharge from hospital (up to six weeks following discharge), for example, blood loss or anaemia that requires treatment, an infection that requires. This also includes life-threating complications such as organ failure, excessive blood loss requiring transfusion, shock, blood clots or a severe infection (sepsis), retained placenta.	107	81.10%	154	80.60%	Yes	Include
Maternal life-threatening complications after birth	For example, organ failure, excessive blood loss requiring transfusion, shock, blood clots or a severe infection (sepsis), retained placenta, near-misses for mortality and morbidity, complications that may require	122	92.40%	166	88.30%	No	Remove: Include as part of 'Maternal complications after birth'
Length of maternal hospital stay due to medical complications after a stillbirth	The number of days or weeks after a stillbirth that a mother needs to stay in hospital due to medical reasons	38	28.60%	76	40.00%	No	Remove: Limited importance in iCHOOSE study
Maternal postpartum recovery	A mother's physical recovery from the birth (typically the first six weeks after the birth), including healing from stitches, pain, bleeding, hormonal changes, breastmilk production	84	64.10%	144	74.60%	No	Remove: Limited importance in iCHOOSE study
Maternal long term physical complications related to the birth	For example, fistula, prolapse, incontinence	102	77.90%	137	71.40%	No	Remove: Include as part of 'Maternal complications after birth'
Maternal long term physical health outcomes	For example, a mother's new medical diagnosis, type 2 diabetes, cancer, heart disease, stroke, blood clots (thrombosis)	94	71.80%	133	70.40%	No	Remove: Would not always be known in LMIC setting
Maternal death	The death of the mother while pregnant, during or following birth and cause of death	126	94.70%	176	93.60%	Yes	Include

Care experience outcomes							
~~Parents' experience of their care following stillbirth~~ *New Outcome Parents' experience of care and support	Parents' views of their care, before, during and after birth including follow-up care at home. Types of care experiences and support might include communication, support and trust from health care professionals and experiences of shared decision making	126	96.90%	179	93.70%	Yes	Include: Change to 'Parents' experience of care and support'
Parents' experience of communication with care professionals	For example, correct and enough information, being listened to, respectful communication, kindness, being involved in discussion, understanding of care options available to them	124	95.40%	185	96.40%	No	Remove: now combined with ' Parents' experience of care and support'
Parents' experience of support from care professionals	How supported parents feel before, during and after birth for example, support with meeting their baby, making memories (taking photos, holding baby), breastmilk and milk donation, decisions about investigations	127	96.90%	184	95.80%	No	Remove: now combined with ' Parents' experience of care and support'
New outcome Parents' experience of shared decision making	Parents' views of feeling involved and informed in decision making with care professionals, having enough information about choices and options in care, feelings about decisions	122	93.10%	175	91.60%	No	Remove: now combined with ' Parents' experience of care and support'
~~Perceived acknowledgement of parenthood by care professionals~~ *New outcome Perceived acknowledgement of parenthood & baby	Parents feel care professionals recognised them as a mother and/or father, and the baby as a person, for example, naming the baby, caring for the baby	119	91.50%	171	90.00%	Yes	Include: Change to 'Perceived acknowledgement of parenthood & baby'
Perceived acknowledgment of baby by care professionals	Parents feel care professionals recognised the baby as a person, for example, naming the baby, caring for the baby	120	91.60%	178	93.70%	No	Remove: now combined with 'Perceived acknowledgement of parenthood & baby'
Impact of providing stillbirth care on healthcare professionals'	For example, wellbeing, emotional and mental health, job role	105	80.20%	169	88.50%	No	Remove: now combined with 'Perceived acknowledgement of parenthood & baby'

New outcome Trust in healthcare professionals	Parents' amount or perceived trust in healthcare professionals after stillbirth	115	87.80%	171	89.10%	No	Remove: now combined with 'Perceived acknowledgement of parenthood & baby'
Formal complaints or legal action	For example, measurement of the number or proportion of formal written complaints or legal (litigation) cases received indicating dissatisfaction with care, or perceived negligent care	63	48.50%	110	60.10%	No	Remove: Limited importance in iCHOOSE study. Unlikely to be done in LMIC setting
**Investigation outcomes**							
Uptake of medical investigations performed to understand why a baby died	For example, autopsy (post-mortem), examination of placenta, genetic testing, determining whether a blood clotting disorder is present	114	87.70%	176	93.60%	Yes	Include
~~Hospital review carried out by healthcare professionals to help understand why the baby died~~ *New outcome Findings of any medical investigations and cause of death communicated to parents	~~Was a review carried out by the hospital to understand why a baby died, for example, by reviewing hospital notes or using a structured system review~~ Were the findings of any medical investigations to undertand why the baby died communicated to parents.	114	88.40%	177	95.20%	Yes	Include: Change to 'Findings of any medical investigations and cause of death communicated to parents'
Uptake of parental engagement in the hospital review to understand why a baby died	Did parents give feedback about their care for the hospital review to understand why a baby died	102	78.50%	165	89.20%	No	Remove: Would not be routinely done in LMIC setting
Cause of death identified	Was a cause of death identified from the medical investigations to understand why a baby died	120	92.30%	180	95.70%	No	Remove: Would not be routinely done in LMIC setting
~~Parents' understanding of why their baby died~~ *New outcome Perceived	Parents feel care professionals recognised them as a mother and/or father, and the baby as a person, for example, naming the baby, caring for the baby	123	94.60%	176	93.60%	Yes	Include: Change to 'Perceived acknowledgement of parenthood & baby'

acknowledgement of parenthood & baby							
Parents' understanding about stillbirth	Parents' awareness of symptoms and signs related to stillbirth, awareness that stillbirth can happen later in pregnancy, parents' understanding of how to prevent a stillbirth in a subsequent pregnancy	122	93.80%	177	94.70%	No	Remove: now combined with 'Perceived acknowledgement of parenthood & baby'
New outcome Additional counselling or subsequent pregnancy care advice following investigations	Information provided from investigations to inform counselling and advice for subsequent pregnancy management, determination of or change in recurrence risk of stillbirth	116	89.20%	180	95.70%	No	Remove: now combined with 'Perceived acknowledgement of parenthood & baby'
New outcome Length of time investigations into understanding why a baby died continue after a stillbirth	Measurement of the length of time it takes for the post-mortem, test results, hospital review to be communicated to parents	95	73.10%	152	81.70%	No	Remove: Would not be routinely done in LMIC setting
Improvements to care and patient safety	Measurement of improvements to care and safety directly as a result of the stillbirth happening, for example, care improvements to prevent future stillbirths, training of care professionals	121	93.10%	180	96.30%	No	Remove: Would not be routinely done in LMIC setting. Seen more as a process outcome

**Grief**							
~~Overwhelming or complicated grief~~ *New outcome Grief	For example, feelings of grief, difficulty coping, self-blame of parents and family members.	105	81.40%	175	94.60%	Yes	Include: Change to 'Grief'
Coping with grief	For example, difficulty coping, managing short and long term grief, coping with key milestones and anniversaries	104	80.60%	173	93.50%	No	Remove: now combined with 'Grief'
Feelings of self-blame, guilt or failure	Parents feeling self-blame, guilty or like a failure as a result of stillbirth and grief	102	79.10%	169	91.40%	No	Remove: now combined with 'Grief'
Perceived acknowledgement of grief by others	Parents feel grief is recognised by family, friends and society	100	77.50%	160	87.40%	No	Remove: now combined with 'Grief'
Grief of whole family	For example, family coping with grief, grief of siblings, children, grandparents or other immediate family members	92	71.90%	149	80.50%	No	Remove: now combined with 'Grief'
**Mental health outcomes**							
~~Depression~~ *New outcome Mental Health and Emotional Wellbeing	Measurement of depressive symptoms, anxiety symptoms, mental health difficulties (for example eating disorders, self-harm, forgetfullness), drug and alcohol use or postitive and negative emotions such as happiness or pessimism.	105	82.70%	159	87.40%	Yes	Include: Change to 'Mental Health and Emotional Wellbeing'
Anxiety	Measurement of anxiety symptoms or medical diagnosis of anxiety disorder, recurrence or worsening of previous anxiety	100	78.70%	160	87.90%	No	Remove: now combined with 'Mental Health and Emotional Wellbeing'
Post-traumatic stress disorder	Measurement of symptoms of trauma or medical diagnosis of post-traumatic stress disorder	108	86.40%	169	93.90%	No	Remove: now combined with 'Mental Health and Emotional Wellbeing'
Suicidal thoughts, attempted suicide, suicide	For example, a parent thinking about or making plans to end their life, a parent takes their own life	114	90.50%	164	91.60%	No	Remove: now combined with 'Mental Health and Emotional Wellbeing'
Other mental health difficulties	For example, psychosis, phobias, eating disorders, obsessive-compulsive disorder (OCD), mental breakdown, self-harm, bipolar disorder	98	77.20%	153	84.50%	No	Remove: now combined with 'Mental Health and Emotional Wellbeing'

Drug and alcohol use	For example excessive alcohol, legal or illegal drug use, drug or alcohol abuse following stillbirth	89	70.60%	128	71.50%	No	Remove: now combined with 'Mental Health and Emotional Wellbeing'
Mental functioning	For example, impact on memory, forgetfulness, concentration, difficulty planning or organising	92	73.60%	147	81.70%	No	Remove: now combined with 'Mental Health and Emotional Wellbeing'
Any mental health treatment (including type)	For example, a parent starts medication or psychological therapy, counselling or alternative therapies such as mindfulness, yoga, meditation	92	72.40%	152	84.00%	No	Remove: now combined with 'Mental Health and Emotional Wellbeing'
**Emotional outcomes**							
Emotional wellbeing	For example, a parents' positive or negative emotions such as happiness or pessimism, emotional resilience or strength	90	72.60%	143	80.30%	No	Remove: now combined with 'Mental Health and Emotional Wellbeing'
New outcome Self-compassion	Parents' feeling caring, kind, and empathetic to them themselves	74	59.70%	127	71.30%	No	Remove: Not something considered in LMIC setting. Less support from parents in iCHOOSE
Self esteem	A parents' feeling of self-worth, for example, positive or negative beliefs about self, self-confidence	82	66.10%	128	71.90%	No	Remove: Not something considered in LMIC setting. Less support from parents in iCHOOSE
Body confidence	How a parent feels about their body and the way they look, for example, feeling attractive and confident in their body, trust in their body	33	26.60%	62	35.00%	No	Remove: Limited importance in iCHOOSE study
Sense of control	The extent of control a parent feels over their life and what happens to them	74	59.70%	114	64.40%	No	Remove: Limited importance in iCHOOSE study
Stress	The degree of physical or emotional strain a parent experiences, high or low stress levels	84	67.70%	133	74.70%	No	Remove: Not something considered in LMIC setting. Less support from parents in iCHOOSE
Sexual wellbeing	For example, sexual functioning and confidence	31	25.20%	52	29.50%	No	Remove: Limited importance in iCHOOSE study
New outcome Mindfulness	The extent a parent able to maintain awareness of the present moment	43	35.80%	83	46.90%	No	Remove: Limited importance in iCHOOSE study

**Whole person outcomes**							
Impact on identity	Impact on the way parents think about themselves and sense of who they are in the world or community, feeling like a parent, mother or father	57	46.00%	110	63.20%	No	Remove: Limited importance in iCHOOSE study
Adjustment to new normal	For example, finding a 'new normal' in life, returning to normal life, activities or role	64	51.60%	124	70.90%	No	Remove: Limited importance in iCHOOSE study
Personal growth or positive impact	For example, positive psychological impact, appreciation of life, increased compassion for others, helping others by volunteering or taking part in research	34	27.40%	62	35.60%	No	Remove: Limited importance in iCHOOSE study
Impact on spirituality	Impact on parents' spiritual beliefs or religion	28	22.60%	32	18.60%	No	Remove: Limited importance in iCHOOSE study
Physical wellbeing	For example, being able to perform normal daily activities required to meet basic needs (e.g. washing, eating and dressing), impact on weight, exercise, tiredness, sleep, physical symptoms of grief	77	62.10%	114	65.50%	No	Remove: Limited importance in iCHOOSE study
Quality of life	For example, life satisfaction related to physical, mental, emotional and physical functioning	89	71.80%	123	70.70%	Yes	Include
**Social outcomes**							
Social impact	For example, difficulty with or avoiding social situations, impact on social life, finding conversations difficult, positive social impact	60	48.40%	112	64.40%	Yes	Include
Opportunities to talk about stillbirth experience with others	Parents feeling they have opportunities to talk about stillbirth and grief with partner, friends, family, strangers, feeling like stillbirth is not a taboo subject	93	75.00%	149	85.60%	Yes	Include
Degree of isolation	For example, feeling alone or abandoned, loneliness, isolated from friends, family and wider society, staying at home, avoiding parents	89	71.80%	142	82.10%	Yes	Include
Perceived stigma from community	For example, parents feel stillbirth viewed negatively by others, disapproval, feeling shame, embarrassment or being judged by community or society	92	74.20%	123	71.50%	Yes	Include

Impact on work	For example, impact on job, being able to do job, unemployment, change of career, workplace discrimination	80	64.50%	131	75.30%	Yes	Include
**Relationship and support outcomes**							
~~Impact on relationship with partner~~ *New Outcome Impact on relationship and perceived support from partner and close family	For example, positive or negative relationship impact, whether parents stay together or not after a stillbirth, needing relationship support, impact on sexual relationship.	98	79.70%	135	78.50%	Yes	Include: Change to 'Impact on relationship and perceived support from partner and close family'
Perceived support from partner	How supported a parent feels by their partner (if applicable), including emotional support	86	69.90%	133	76.90%	No	Remove: now combined 'Impact on relationship and perceived support from partner and close family'
Impact on relationships with family, friends and community	For example, positive or negative impact on relationships, loss of friendships or new friendships	84	68.30%	116	67.10%	No	Remove: now combined 'Impact on relationship and perceived support from partner and close family'
Perceived support from family, friends and community	How supported a parent feels by their family members, friends and community, including spiritual and emotional support	68	55.30%	107	61.80%	No	Remove: now combined 'Impact on relationship and perceived support from partner and close family'
Perceived support with returning to work	How supported a parent feels with returning to work, for example, workplace offers enough time off work, parental leave, support telling work colleagues, phased return to work, access to counselling or occupational therapy through work	70	56.90%	119	68.80%	No	Remove: now combined with 'Impact on work'
Satisfaction with support resources and support groups	For example, books, leaflets, social media, online resources, tv programmes, positive and negative experiences, charity support groups, positive and negative experiences	79	64.20%	120	69.40%	No	Remove: Not something always considered in LMIC setting. More of a process otucome.
**Older children outcomes**							
Psychological health of older children in the family	For example, short-term and long-term mental and emotional health, impact on behaviour and school	83	68.00%	74	88.10%	No	Remove: now combined 'Impact on relationship and perceived support from partner and close family'

Impact on parenting	For example, more protective about older children or more anxious, parents more distant with older children	87	71.90%	72	86.70%	No	Remove: now combined 'Impact on relationship and perceived support from partner and close family'
Support for older children and parenting	For example, support with talking about stillbirth with older children in the family, help and support from school, charity support, counselling	76	62.30%	65	77.40%	No	Remove: now combined 'Impact on relationship and perceived support from partner and close family'
**Economic outcomes**							
Financial costs for parents	For example, costs of hospital care (in countries where parents pay for hospital care related to stillbirth), impact on pay from missing work, costs of visiting hospital, costs of funeral or counselling, costs of care in subsequent pregnancies for parents	74	61.20%	97	57.40%	Yes	Include
Financial costs for health service and wider society	For example, additional costs of stillbirth care in short and long term and subsequent pregnancies for healthcare providers, financial costs to society (such as loss of workplace productivity)	50	41.30%	55	32.70%	Yes	Include
Survival of baby/ies after stillbirth is identified in a multiple pregnancy	Livebirth, stillbirth, termination of pregnancy or neonatal death of baby/ies	97	81.50%	25	92.60%	No	Remove: Multiple-pregnancies is seen as out of scope for this COS (possibly addresses a different research question)

**Twin or multiple outcomes**							
Preterm birth of surviving baby(ies) after stillbirth is identified in a multiple pregnancy	Surviving baby(ies) born before 37 weeks	90	75.60%	24	92.30%	No	Remove: Multiple-pregnancies is seen as out of scope for this COS (possibly addresses a different research question)
Pregnancy complications that risk the life of the surviving baby(ies) after stillbirth is identified in a multiple pregnancy	For example, growth restriction, fetal anaemia, requiring intrauterine transfusion, premature labour	98	80.30%	27	96.40%	No	Remove: Multiple-pregnancies is seen as out of scope for this COS (possibly addresses a different research question)
Pregnancy complications for the mother after stillbirth is identified in a multiple pregnancy	For example, infection, blood clotting problems, pre-eclampsia	95	77.90%	25	92.60%	No	Remove: Multiple-pregnancies is seen as out of scope for this COS (possibly addresses a different research question)
Neonatal outcomes of surviving baby(ies) after stillbirth is identified in a multiple pregnancy	Newborn outcomes e.g. admission to neonatal intensive care unit, respiratory distress, Apgar score, birth weight	100	82.00%	26	96.30%	No	Remove: Multiple-pregnancies is seen as out of scope for this COS (possibly addresses a different research question)
Neurodevelopment of surviving baby(ies) after stillbirth is identified in a multiple pregnancy	For example, whether the surviving baby(ies) reaches their developmental milestones, brain injury	91	75.20%	26	96.30%	No	Remove: Multiple-pregnancies is seen as out of scope for this COS (possibly addresses a different research question)
Medical health of surviving baby(ies) after stillbirth is identified in a multiple pregnancy	For example, whether the surviving baby(ies) has any medical or genetic problems, monitoring of surviving multiple, concern about health	87	71.30%	25	92.60%	No	Remove: Multiple-pregnancies is seen as out of scope for this COS (possibly addresses a different research question)
Attachment to surviving baby(ies) after stillbirth is identified in a multiple pregnancy	Parental connection or bonding with surviving baby(ies)	91	75.20%	22	78.60%	No	Remove: Multiple-pregnancies is seen as out of scope for this COS (possibly addresses a different research question)

Psychological health of surviving child after stillbirth is identified in a multiple pregnancy	For example, short-term and long-term mental and emotional health, need for psychological support, counselling, support from school	72	59.50%	18	64.30%	No	Remove: Multiple-pregnancies is seen as out of scope for this COS (possibly addresses a different research question)
**Planning subsequent pregnancy outcomes**							
Perceived support for planning next pregnancy after stillbirth	How supported parents feel with planning next pregnancy, risk factors addressed, health optimised to prevent future stillbirth, advice given on timing of a pregnancy, contraception advice, support with choosing not to become pregnant	99	81.10%	148	87.10%	Yes	Include - seen as important to have one outcome on subsequent pregnancies.
Need for fertility treatment after stillbirth	Help needed from healthcare professionals to become pregnant, for example, fertility tests or IV	44	36.40%	95	58.30%	No	Remove: Limited importance LB study (even parents), not overwhelming from think aloud
Infertility	A couple cannot conceive again after having regular unprotected sex for more than twelve months	45	37.20%	96	58.50%	No	Remove: Limited importance LB study (even parents), not overwhelming from think aloud
Parents choosing not to become pregnant again after a stillbirth	Parents deciding not to have another baby	45	37.20%	58	36.00%	No	Remove: Limited importance LB study (even parents), not overwhelming from think aloud
**Subsequent pregnancy outcomes**							
Time between stillbirth and next pregnancy	Amount of time between stillbirth pregnancy and next pregnancy	54	44.60%	64	51.20%	No	Remove: Subsequent pregnancies is seen as out of scope for this COS (possibly addresses a different research question)
Number of pregnancies between stillbirth and live birth	Number of pregnancies after a stillbirth before live birth happens	65	53.70%	83	67.50%	No	Remove: Subsequent pregnancies is seen as out of scope for this COS (possibly addresses a different research question)
New outcome Conception rate	How many people become pregnant after stillbirth	51	42.10%	75	61.00%	No	Remove: Subsequent pregnancies is seen as out of scope for this COS (possibly addresses a different research question)
Complications for the baby in a subsequent pregnancy after stillbirth	For example, growth restriction, genetic or congenital anomaly	98	81.00%	118	94.40%	No	Remove: Subsequent pregnancies is seen as out of scope for this COS (possibly addresses a different research question)

Complications for the mother in a subsequent pregnancy after stillbirth	For example, bleeding, obstetric cholestasis, low lying placenta, premature rupture of membranes, gestational diabetes, pre-eclampsia	96	79.30%	116	92.80%	No	Remove: Subsequent pregnancies is seen as out of scope for this COS (possibly addresses a different research question)
Preterm birth in a subsequent pregnancy after stillbirth	Next baby born before 37 weeks' gestation	86	71.10%	109	87.90%	No	Remove: Subsequent pregnancies is seen as out of scope for this COS (possibly addresses a different research question)
Induction of labour in a subsequent pregnancy after stillbirth	Whether a mother was induced in a subsequent pregnancy and at what number of weeks	60	50.00%	83	66.40%	No	Remove: Subsequent pregnancies is seen as out of scope for this COS (possibly addresses a different research question)
Type of birth in a subsequent pregnancy after stillbirth	Caesarean section, spontaneous vaginal, assisted vaginal birth (forceps or ventouse)	52	43.30%	64	51.20%	No	Remove: Limited importance LB study (even parents), not overwhelming from think aloud
Birth and postpartum complications in a subsequent pregnancy after stillbirth	For example, shoulder dystocia, obstructed labour, postpartum bleeding, retained placenta	73	60.80%	97	78.90%	No	Remove: Subsequent pregnancies is seen as out of scope for this COS (possibly addresses a different research question)
Survival of baby in a subsequent pregnancy after stillbirth	Live birth, miscarriage, stillbirth, termination of pregnancy, neonatal death of baby in a subsequent pregnancy	107	89.20%	118	95.20%	No	Remove: Subsequent pregnancies is seen as out of scope for this COS (possibly addresses a different research question)
Newborn outcomes in baby born after stillbirth	Newborn outcomes, for example Apgar score, birth weight of baby, admission to neonatal intensive care unit, gestational age	93	77.50%	110	88.00%	No	Remove: Subsequent pregnancies is seen as out of scope for this COS (possibly addresses a different research question)
Additional scans and clinic appointments during subsequent pregnancy after stillbirth	Parents getting additional ultrasound scans during the pregnancy to check wellbeing of the baby	92	76.00%	117	93.60%	No	Remove: Subsequent pregnancies is seen as out of scope for this COS (possibly addresses a different research question)
Unplanned hospital admission prior to birth of baby in a subsequent pregnancy after stillbirth	Admission to hospital to monitor baby, staying in hospital until the birth, due to medical complications or to offer reassurance to parents	73	60.30%	102	82.30%	No	Remove: Subsequent pregnancies is seen as out of scope for this COS (possibly addresses a different research question)
Perceived support for subsequent pregnancy,	How prepared parents feel for subsequent pregnancy, birth and baby, specialist antenatal clinic or classes	92	76.00%	110	88.00%	No	Remove: Subsequent pregnancies is seen as out of scope for this COS

birth and parenthood after stillbirth							(possibly addresses a different research question)
Coping in a subsequent pregnancy after stillbirth	Coping with fears about pregnancy, childbirth, survival of baby, health of baby or parenthood	104	86.00%	115	92.00%	No	Remove: Subsequent pregnancies is seen as out of scope for this COS (possibly addresses a different research question)
New outcome Anxiety related to subsequent pregnancy and children	Specific anxiety as a result of a subsequent pregnancy and children	100	82.60%	116	92.80%	No	Remove: Subsequent pregnancies is seen as out of scope for this COS (possibly addresses a different research question)
Attachment to baby during subsequent pregnancy after stillbirth	Connection or bonding with new baby during subsequent pregnancy or acceptance of pregnancy	95	78.50%	110	87.30%	No	Remove: Subsequent pregnancies is seen as out of scope for this COS (possibly addresses a different research question)
Parents' satisfaction with care in a subsequent pregnancy after stillbirth	Parents' satisfaction with specialist antenatal clinic, bereavement care, specialist postnatal care with breastfeeding	96	79.30%	111	88.80%	No	Remove: Subsequent pregnancies is seen as out of scope for this COS (possibly addresses a different research question)
**Subsequent children outcomes**							
Development of child born after a stillbirth	Whether a child is reaching developmental milestones such as speech, thinking and movement	16	13.30%	26	28.00%	No	Remove: Subsequent children is seen as out of scope for this COS (possibly addresses a different research question)
Medical health of child born after stillbirth	Short- or long-term medical health of subsequent child, genetic condition	23	19.20%	29	31.50%	No	Remove: Subsequent children is seen as out of scope for this COS (possibly addresses a different research question)
Attachment to a child born after stillbirth	Bonding or connection with a subsequent child	69	57.50%	63	67.70%	No	Remove: Subsequent children is seen as out of scope for this COS (possibly addresses a different research question)
Psychological health of child born after stillbirth	For example, emotional or mental health, need for psychological support, counselling or help from school as a result of stillbirth	46	38.30%	49	52.70%	No	Remove: Subsequent children is seen as out of scope for this COS (possibly addresses a different research question)
