# Supplementary material for: A meta-core outcome set for stillbirth prevention and bereavement care following stillbirth in LMIC
Source: BMJ Glob Health. 2025 Jan 28;10(1):e017688. doi: 10.1136/bmjgh-2024-017688 (PMC11781104; doi:10.1136/bmjgh-2024-017688)
Supplement: online supplemental file 3 [file bmjgh-10-1-s003.pdf]

Supplementary Table 2a: Final real-time Delphi survey results by stakeholder group: stillbirth prevention.

Prevention

	Healthcare Professionals (Obstetrician)																			Percentage 7-9
	N	1	%	2	%	3	%	4	%	5	%	6	%	7	%	8	%	9	%	
**Obstetric Outcomes**																				
Spontaneous preterm birth	32	0	0%	0	0%	1	3%	1	3%	1	3%	1	3%	3	9%	6	19%	19	59%	88%
Mode of delivery	32	0	0%	1	3%	0	0%	0	0%	3	9%	3	9%	5	16%	2	6%	18	56%	78%
Hypertension	32	0	0%	0	0%	0	0%	0	0%	1	3%	1	3%	2	6%	3	9%	25	78%	94%
Timing of stillbirth (antepartum or intrapartum)	32	0	0%	0	0%	0	0%	0	0%	3	9%	0	0%	1	3%	7	22%	21	66%	91%
Multiple gestational pregnancies	32	0	0%	0	0%	0	0%	2	6%	1	3%	3	9%	6	19%	9	28%	11	34%	81%
**Fetal Outcomes**																				
Maternal reports of reduced foetal movements	30	0	0%	0	0%	0	0%	0	0%	1	3%	0	0%	6	20%	5	17%	18	60%	97%
Signs of compromise requiring emergency birth	30	0	0%	0	0%	0	0%	0	0%	1	3%	1	3%	1	3%	6	20%	21	70%	93%
Harm to fetus from intervention	30	0	0%	0	0%	2	7%	0	0%	2	7%	1	3%	8	27%	6	20%	11	37%	83%
**Perinatal Outcomes**																				
Stillbirth	31	0	0%	0	0%	0	0%	0	0%	2	6%	0	0%	2	3%	3	24%	24	77%	94%
Perinatal mortality	31	0	0%	0	0%	0	0%	0	0%	0	0%	0	0%	2	6%	3	10%	26	84%	100%
Neonatal mortality	30	0	0%	1	3%	0	0%	0	0%	0	0%	2	7%	2	7%	5	17%	20	67%	90%
Birthweight	31	0	0%	0	0%	0	0%	0	0%	1	3%	1	3%	2	6%	5	16%	22	71%	94%
Small for gestational age	31	0	0%	0	0%	0	0%	0	0%	0	0%	0	0%	3	10%	8	26%	20	65%	100%
Miscarriage	31	2	6%	0	0%	1	3%	0	0%	5	16%	1	3%	5	16%	3	10%	14	45%	71%
Apgar <7 at 5 minutes	30	0	0%	0	0%	1	3%	1	3%	0	0%	2	7%	3	10%	2	7%	21	70%	87%
Gestational Diabetes Mellitus	30	0	0%	0	0%	0	0%	0	0%	0	0%	0	0%	4	13%	6	20%	20	67%	100%
Gestational Age at Diagnosis of Stillbirth	31	0	0%	0	0%	0	0%	0	0%	0	0%	2	6%	2	6%	8	26%	19	61%	94%

**Maternal Complications**																				
Eclampsia	30	0	0%	0	0%	0	0%	0	0%	0	0%	0	0%	2	7%	4	13%	24	80%	100%
Placental abruption	30	0	0%	0	0%	0	0%	0	0%	0	0%	1	3%	0	0%	6	20%	23	77%	97%
Chorioamnionitis	30	0	0%	0	0%	0	0%	0	0%	0	0%	2	7%	5	17%	5	17%	18	60%	93%
Harm to mother from intervention	29	1	3%	0	0%	0	0%	1	3%	1	3%	3	10%	5	17%	7	24%	11	38%	79%
Antepartum haemorrhage	30	0	0%	0	0%	0	0%	0	0%	0	0%	1	3%	1	3%	5	17%	23	77%	97%
Postpartum haemorrhage	28	1	4%	3	11%	3	11%	5	18%	1	4%	2	7%	2	7%	2	7%	9	32%	46%
**Maternal Outcomes**																				
Antenatal/postnatal depression	29	0	0%	0	0%	1	3%	0	0%	3	10%	3	10%	9	31%	3	10%	10	34%	76%
Maternal mortality	28	0	0%	0	0%	0	0%	0	0%	2	7%	0	0%	6	21%	3	11%	17	61%	93%
Social isolation	28	0	0%	1	4%	1	4%	0	0%	5	18%	1	4%	3	11%	7	25%	10	36%	71%
Childbirth experience	30	0	0%	0	0%	0	0%	0	0%	0	0%	7	23%	2	7%	4	13%	17	57%	77%
Women's knowledge	30	0	0%	0	0%	0	0%	0	0%	0	0%	3	10%	2	7%	7	23%	18	60%	90%
Women's nutritional status	30	0	0%	0	0%	0	0%	0	0%	2	7%	4	13%	4	13%	8	27%	12	40%	80%
**Neonatal Complication**																				
Intraventricular haemorrhage	28	0	0%	1	4%	1	4%	0	0%	1	4%	4	14%	7	25%	2	7%	12	43%	75%
Neonatal seizures	29	1	3%	0	0%	2	7%	0	0%	0	0%	2	7%	6	21%	4	14%	14	48%	83%
Respiratory distress syndrome	29	1	3%	1	3%	1	3%	0	0%	1	3%	2	7%	4	14%	4	14%	15	52%	79%
Neonatal infection	29	1	3%	0	0%	1	3%	0	0%	1	3%	2	7%	3	10%	3	10%	18	62%	83%
**Neonatal Outcome**																				
Gestational age	28	0	0%	0	0%	0	0%	0	0%	0	0%	2	7%	2	7%	9	32%	15	54%	93%
Neonatal intensive care unit (NICU) admission	27	0	0%	0	0%	0	0%	0	0%	1	4%	1	4%	2	7%	8	30%	15	56%	93%
Congenital anomaly	28	0	0%	0	0%	0	0%	0	0%	1	4%	0	0%	4	14%	4	14%	19	68%	96%
**Health Service Outcome**																				
Number of antenatal visits	28	0	0%	0	0%	0	0%	0	0%	1	4%	4	14%	4	14%	5	18%	14	50%	82%
NICU/Special Care Baby Unit length of stay (days)	27	0	0%	0	0%	3	11%	0	0%	1	4%	2	7%	4	15%	3	11%	14	52%	78%
**Other**																				
Stigma	27	0	0%	2	7%	3	11%	0	0%	3	11%	5	19%	3	11%	3	11%	8	30%	52%

Prevention

	Healthcare Professionals (Nurse Midwives)																			
	N	1	%	2	%	3	%	4	%	5	%	6	%	7	%	8	%	9	%	Percentage 7-9
**Obstetric Outcomes**																				
Spontaneous preterm birth	125	1	1%	0	0%	3	2%	2	2%	8	6%	2	2%	7	6%	22	18%	80	64%	87%
Mode of delivery	123	2	2%	2	2%	4	3%	5	4%	6	5%	4	3%	6	5%	22	18%	72	59%	81%
Hypertension	123	0	0%	0	0%	0	0%	1	1%	2	2%	3	2%	4	3%	12	10%	101	82%	95%
Timing of stillbirth (antepartum or intrapartum)	114	2	2%	2	2%	1	1%	4	4%	4	4%	3	3%	7	6%	24	21%	67	59%	86%
Multiple gestational pregnancies	118	2	2%	0	0%	2	2%	3	3%	8	7%	10	8%	4	3%	18	15%	71	60%	79%
**Foetal Outcomes**																				
Maternal reports of reduced foetal movements	111	0	0%	0	0%	0	0%	0	0%	4	4%	0	0%	4	4%	12	11%	91	82%	96%
Signs of compromise requiring emergency birth	112	0	0%	1	1%	0	0%	1	1%	3	3%	4	4%	2	2%	15	13%	86	77%	92%
Harm to foetus from intervention	110	1	1%	3	3%	2	2%	3	3%	5	5%	4	4%	9	8%	21	19%	62	56%	84%
**Perinatal Outcomes**																				
Stillbirth	103	0	0%	0	0%	2	2%	2	2%	3	3%	7	7%	5	5%	12	12%	72	70%	86%
Perinatal mortality	103	2	2%	0	0%	2	2%	3	3%	8	8%	4	4%	4	4%	12	12%	68	66%	82%
Neonatal mortality	102	5	5%	1	1%	1	1%	4	4%	7	7%	5	5%	2	2%	16	16%	61	60%	77%
Birthweight	102	4	4%	3	3%	1	1%	2	2%	3	3%	2	2%	7	7%	22	22%	58	57%	85%
Small for gestational age	99	1	1%	2	2%	0	0%	4	4%	5	5%	2	2%	7	7%	22	22%	56	57%	86%
Miscarriage	98	5	5%	1	1%	1	1%	2	2%	6	6%	6	6%	11	11%	12	12%	54	55%	79%
Apgar <7 at 5 minutes	101	5	5%	2	2%	1	1%	2	2%	0	0%	5	5%	6	6%	10	10%	70	69%	85%
Gestational Diabetes Mellitus	102	0	0%	0	0%	0	0%	4	4%	5	5%	1	1%	8	8%	14	14%	70	69%	90%
Gestational Age at Diagnosis of Stillbirth	101	1	1%	1	1%	1	1%	2	2%	5	5%	4	4%	9	9%	14	14%	64	63%	86%

**Maternal Complications**																			
Eclampsia	104	0	0%	1	1%	0	0%	0	0%	2	2%	3	3%	3	3%	8	8%	87	84%	94%
Placental abruption	101	0	0%	0	0%	1	1%	0	0%	2	2%	3	3%	5	5%	12	12%	78	77%	94%
Chorioamnionitis	102	0	0%	1	1%	0	0%	0	0%	10	10%	5	5%	7	7%	13	13%	66	65%	84%
Harm to mother from intervention	103	5	5%	1	1%	1	1%	7	7%	8	8%	10	10%	8	8%	13	13%	50	49%	69%
Antepartum haemorrhage	102	0	0%	0	0%	0	0%	0	0%	1	1%	3	3%	6	6%	10	10%	82	80%	96%
Postpartum haemorrhage	103	17	17%	5	5%	4	4%	4	4%	13	13%	7	7%	3	3%	8	8%	42	41%	51%
**Maternal Outcomes**																			
Antenatal/postnatal depression	99	5	5%	2	2%	2	2%	4	4%	9	9%	11	11%	10	10%	15	15%	41	41%	67%
Maternal mortality	99	4	4%	4	4%	1	1%	5	5%	5	5%	6	6%	11	11%	10	10%	53	54%	75%
Social isolation	99	4	4%	1	1%	6	6%	4	4%	15	15%	9	9%	10	10%	17	17%	33	33%	61%
Childbirth experience	97	3	3%	3	3%	1	1%	1	1%	11	11%	6	6%	15	15%	21	22%	36	37%	74%
Women's knowledge	97	0	0%	1	1%	0	0%	2	2%	8	8%	3	3%	10	10%	17	18%	56	58%	86%
Women's nutritional status	98	0	0%	0	0%	1	1%	3	3%	5	5%	5	5%	12	12%	16	16%	56	57%	86%
**Neonatal Complication**																			
Intraventricular haemorrhage	93	3	3%	1	1%	2	2%	1	1%	6	6%	6	6%	6	6%	12	13%	56	60%	80%
Neonatal seizures	97	6	6%	2	2%	3	3%	3	3%	2	2%	2	2%	6	6%	13	13%	60	62%	81%
Respiratory distress syndrome	97	7	7%	2	2%	2	2%	2	2%	3	3%	3	3%	1	1%	14	14%	63	65%	80%
Neonatal infection	96	10	10%	1	1%	2	2%	3	3%	2	2%	2	2%	6	6%	17	18%	53	55%	79%
**Neonatal Outcome**																			
Gestational age	96	1	1%	1	1%	1	1%	2	2%	3	3%	4	4%	10	10%	12	13%	62	65%	88%
Neonatal intensive care unit (NICU) admission	96	6	6%	1	1%	0	0%	1	1%	4	4%	1	1%	6	6%	10	10%	67	70%	86%
Congenital anomaly	95	1	1%	2	2%	1	1%	0	0%	3	3%	6	6%	6	6%	13	14%	63	66%	86%
**Health Service Outcome**																			
Number of antenatal visits	96	0	0%	2	2%	0	0%	3	3%	5	5%	3	3%	5	5%	20	21%	58	60%	86%
NICU/Special Care Baby Unit length of stay (days)	94	6	6%	1	1%	3	3%	3	3%	6	6%	5	5%	7	7%	13	14%	50	53%	74%
**Other**																			
Stigma	94	7	7%	1	1%	6	6%	2	2%	6	6%	10	11%	13	14%	19	20%	30	32%	66%

Prevention

	Parents																			
	N	1	%	2	%	3	%	4	%	5	%	6	%	7	%	8	%	9	%	Percentage 7-9
**Obstetric Outcomes**																				
Spontaneous preterm birth	61	1	2%	0	0%	0	0%	0	0%	1	2%	0	0%	5	8%	9	15%	45	74%	97%
Mode of delivery	60	0	0%	0	0%	1	2%	1	2%	1	2%	4	7%	6	10%	11	18%	36	60%	88%
Hypertension	58	0	0%	0	0%	1	2%	0	0%	1	2%	1	2%	4	7%	14	24%	37	64%	95%
Timing of stillbirth (antepartum or intrapartum)	59	1	2%	1	2%	0	0%	0	0%	1	2%	4	7%	6	10%	13	22%	33	56%	88%
Multiple gestational pregnancies	60	0	0%	1	2%	0	0%	0	0%	3	5%	2	3%	3	5%	13	22%	38	63%	90%
**Foetal Outcomes**																				
Maternal reports of reduced foetal movements	58	2	3%	1	2%	0	0%	1	2%	2	3%	6	10%	3	5%	6	10%	37	64%	79%
Signs of compromise requiring emergency birth	57	1	2%	0	0%	0	0%	5	9%	0	0%	0	0%	5	9%	6	11%	40	70%	89%
Harm to foetus from intervention	55	0	0%	0	0%	0	0%	1	2%	4	7%	2	4%	6	11%	13	24%	29	53%	87%
**Perinatal Outcomes**																				
Stillbirth	55	2	4%	2	4%	0	0%	1	2%	2	4%	1	2%	3	5%	11	20%	33	60%	85%
Perinatal mortality	56	2	4%	1	2%	2	4%	2	4%	0	0%	5	9%	0	0%	6	11%	38	68%	79%
Neonatal mortality	55	2	4%	2	4%	0	0%	0	0%	0	0%	6	11%	4	7%	9	16%	32	58%	82%
Birthweight	56	2	4%	1	2%	1	2%	0	0%	3	5%	7	13%	2	4%	11	20%	29	52%	75%
Small for gestational age	55	1	2%	0	0%	0	0%	3	5%	2	4%	2	4%	4	7%	9	16%	34	62%	85%
Miscarriage	53	0	0%	1	2%	1	2%	2	4%	3	6%	5	9%	4	8%	4	8%	33	62%	77%
Apgar <7 at 5 minutes	55	1	2%	0	0%	0	0%	2	4%	5	9%	1	2%	2	4%	8	15%	36	65%	84%
Gestational Diabetes Mellitus	56	1	2%	2	4%	1	2%	0	0%	2	4%	1	2%	6	11%	6	11%	37	66%	88%
Gestational Age at Diagnosis of Stillbirth	55	1	2%	2	4%	2	4%	1	2%	2	4%	1	2%	2	4%	13	24%	31	56%	84%

**Maternal Complications**																				
Eclampsia	55	0	0%	1	2%	2	4%	0	0%	0	0%	2	4%	3	5%	8	15%	39	71%	91%
Placental abruption	55	0	0%	2	4%	0	0%	1	2%	1	2%	0	0%	3	5%	13	24%	35	64%	93%
Chorioamnionitis	55	1	2%	0	0%	0	0%	0	0%	1	2%	2	4%	6	11%	8	15%	37	67%	93%
Harm to mother from intervention	54	1	2%	1	2%	1	2%	0	0%	1	2%	2	4%	10	19%	7	13%	31	57%	89%
Antepartum haemorrhage	54	0	0%	1	2%	1	2%	2	4%	2	4%	3	6%	1	2%	7	13%	37	69%	83%
Postpartum haemorrhage	56	3	5%	1	2%	1	2%	2	4%	0	0%	2	4%	2	4%	6	11%	39	70%	84%
**Maternal Outcomes**																				
Antenatal/postnatal depression	55	1	2%	1	2%	2	4%	0	0%	5	9%	2	4%	6	11%	8	15%	30	55%	80%
Maternal mortality	55	1	2%	1	2%	0	0%	2	4%	0	0%	3	5%	6	11%	8	15%	34	62%	87%
Social isolation	55	2	4%	0	0%	0	0%	1	2%	5	9%	5	9%	5	9%	12	22%	25	45%	76%
Childbirth experience	55	0	0%	0	0%	0	0%	3	5%	3	5%	4	7%	6	11%	10	18%	29	53%	82%
Women's knowledge	55	1	2%	0	0%	0	0%	0	0%	5	9%	0	0%	5	9%	10	18%	34	62%	89%
Women's nutritional status	54	0	0%	0	0%	0	0%	1	2%	2	4%	2	4%	7	13%	5	9%	37	69%	91%
**Neonatal Complication**																				
Intraventricular haemorrhage	55	0	0%	0	0%	1	2%	0	0%	3	5%	3	5%	3	5%	8	15%	37	67%	87%
Neonatal seizures	55	1	2%	1	2%	0	0%	2	4%	1	2%	5	9%	1	2%	8	15%	36	65%	82%
Respiratory distress syndrome	55	1	2%	1	2%	0	0%	0	0%	0	0%	2	4%	4	7%	4	7%	43	78%	93%
Neonatal infection	54	3	6%	0	0%	0	0%	0	0%	2	4%	2	4%	2	4%	8	15%	37	69%	87%
**Neonatal Outcome**																				
Gestational age	54	1	2%	0	0%	0	0%	0	0%	4	7%	1	2%	7	13%	8	15%	33	61%	89%
Neonatal intensive care unit (NICU) admission	53	2	4%	0	0%	0	0%	0	0%	3	6%	0	0%	2	4%	11	21%	35	66%	91%
Congenital anomaly	54	3	6%	1	2%	3	6%	2	4%	3	6%	1	2%	3	6%	5	9%	33	61%	76%
**Health Service Outcome**																				
Number of antenatal visits	54	0	0%	0	0%	1	2%	2	4%	1	2%	4	7%	2	4%	11	20%	33	61%	85%
NICU/Special Care Baby Unit length of stay (days)	53	2	4%	0	0%	1	2%	1	2%	2	4%	4	8%	6	11%	9	17%	28	53%	81%
**Other**																				
Stigma	53	3	6%	1	2%	1	2%	4	8%	1	2%	4	8%	1	2%	10	19%	28	53%	74%

Prevention

	Researchers																			
	N	1	%	2	%	3	%	4	%	5	%	6	%	7	%	8	%	9	%	Percentage 7-9
**Obstetric Outcomes**																				
Spontaneous preterm birth	47	1	2%	0	0%	0	0%	0	0%	2	4%	4	9%	6	13%	9	19%	25	53%	85%
Mode of delivery	47	0	0%	1	2%	1	2%	3	6%	1	2%	1	2%	7	15%	12	26%	21	45%	85%
Hypertension	47	0	0%	0	0%	0	0%	0	0%	0	0%	7	15%	5	11%	10	21%	25	53%	85%
Timing of stillbirth (antepartum or intrapartum)	47	0	0%	0	0%	1	2%	0	0%	5	11%	5	11%	3	6%	13	28%	20	43%	77%
Multiple gestational pregnancies	46	0	0%	0	0%	0	0%	1	2%	4	9%	5	11%	7	15%	11	24%	18	39%	78%
**Fetal Outcomes**																				
Maternal reports of reduced fetal movements	46	0	0%	0	0%	2	4%	1	2%	2	4%	4	9%	6	13%	5	11%	26	57%	80%
Signs of compromise requiring emergency birth	45	0	0%	0	0%	0	0%	0	0%	1	2%	6	13%	2	4%	7	16%	29	64%	84%
Harm to fetus from intervention	46	0	0%	0	0%	1	2%	2	4%	5	11%	4	9%	5	11%	6	13%	23	50%	74%
**Perinatal Outcomes**																				
Stillbirth	44	0	0%	0	0%	0	0%	0	0%	1	2%	3	7%	4	9%	9	20%	27	61%	91%
Perinatal mortality	43	0	0%	0	0%	0	0%	1	2%	3	7%	7	16%	4	9%	6	14%	22	51%	74%
Neonatal mortality	45	2	4%	0	0%	2	4%	2	4%	2	4%	2	4%	8	18%	9	20%	18	40%	78%
Birthweight	44	2	5%	0	0%	0	0%	0	0%	3	7%	4	9%	9	20%	9	20%	17	39%	80%
Small for gestational age	45	1	2%	0	0%	1	2%	0	0%	3	7%	9	20%	8	18%	9	20%	14	31%	69%
Miscarriage	45	2	4%	1	2%	1	2%	1	2%	7	16%	4	9%	6	13%	8	18%	15	33%	64%
Apgar <7 at 5 minutes	42	2	5%	0	0%	0	0%	2	5%	6	14%	2	5%	2	5%	10	24%	18	43%	71%
Gestational Diabetes Mellitus	45	0	0%	0	0%	1	2%	0	0%	6	13%	7	16%	7	16%	5	11%	19	42%	69%
Gestational Age at Diagnosis of Stillbirth	44	0	0%	0	0%	0	0%	0	0%	3	7%	8	18%	5	11%	7	16%	21	48%	75%

**Maternal Complications**																				
Eclampsia	44	1	2%	0	0%	0	0%	1	2%	1	2%	2	5%	6	14%	10	23%	23	52%	89%
Placental abruption	44	0	0%	0	0%	1	2%	2	5%	0	0%	3	7%	4	9%	12	27%	22	50%	86%
Chorioamnionitis	43	0	0%	0	0%	1	2%	1	2%	1	2%	7	16%	6	14%	8	19%	19	44%	77%
Harm to mother from intervention	41	0	0%	2	5%	1	2%	1	2%	3	7%	5	12%	9	22%	4	10%	16	39%	71%
Antepartum haemorrhage	44	1	2%	0	0%	1	2%	1	2%	0	0%	3	7%	5	11%	11	25%	22	50%	86%
Postpartum haemorrhage	44	6	14%	2	5%	3	7%	2	5%	5	11%	6	14%	4	9%	4	9%	12	27%	45%
**Maternal Outcomes**																				
Antenatal/postnatal depression	43	3	7%	1	2%	1	2%	1	2%	4	9%	5	12%	8	19%	5	12%	15	35%	65%
Maternal mortality	41	4	10%	0	0%	1	2%	1	2%	3	7%	3	7%	7	17%	4	10%	18	44%	71%
Social isolation	41	4	10%	0	0%	2	5%	1	2%	6	15%	5	12%	8	20%	7	17%	8	20%	56%
Childbirth experience	41	1	2%	0	0%	2	5%	0	0%	3	7%	6	15%	7	17%	9	22%	13	32%	71%
Women's knowledge	42	1	2%	0	0%	1	2%	0	0%	5	12%	4	10%	5	12%	10	24%	16	38%	74%
Women's nutritional status	42	0	0%	0	0%	0	0%	1	2%	4	10%	4	10%	8	19%	9	21%	16	38%	79%
**Neonatal Complication**																				
Intraventricular haemorrhage	42	5	12%	2	5%	0	0%	1	2%	4	10%	4	10%	6	14%	5	12%	15	36%	62%
Neonatal seizures	42	5	12%	2	5%	1	2%	0	0%	4	10%	5	12%	7	17%	4	10%	14	33%	60%
Respiratory distress syndrome	42	7	17%	1	2%	0	0%	1	2%	2	5%	7	17%	5	12%	7	17%	12	29%	57%
Neonatal infection	42	7	17%	0	0%	0	0%	0	0%	5	12%	7	17%	4	10%	3	7%	16	38%	55%
**Neonatal Outcome**																				
Gestational age	41	0	0%	0	0%	1	2%	0	0%	3	7%	5	12%	4	10%	8	20%	20	49%	78%
Neonatal intensive care unit (NICU) admission	40	5	13%	0	0%	1	3%	1	3%	4	10%	6	15%	3	8%	5	13%	15	38%	58%
Congenital anomaly	40	1	3%	0	0%	0	0%	1	3%	4	10%	7	18%	3	8%	4	10%	20	50%	68%
**Health Service Outcome**																				
Number of antenatal visits	39	0	0%	0	0%	0	0%	1	3%	3	8%	6	15%	8	21%	5	13%	16	41%	74%
NICU/Special Care Baby Unit length of stay (days)	40	4	10%	2	5%	0	0%	1	3%	3	8%	9	23%	5	13%	8	20%	8	20%	53%
**Other**																				
Stigma	40	1	3%	0	0%	1	3%	1	3%	7	18%	6	15%	5	13%	6	15%	13	33%	60%
