# Supplementary material for: A meta-core outcome set for stillbirth prevention and bereavement care following stillbirth in LMIC
Source: BMJ Glob Health. 2025 Jan 28;10(1):e017688. doi: 10.1136/bmjgh-2024-017688 (PMC11781104; doi:10.1136/bmjgh-2024-017688)
Supplement: online supplemental file 4 [file bmjgh-10-1-s004.pdf]

Supplementary Table 2b: Final real-time Delphi survey results by stakeholder group: bereavement care following stillbirth.

Bereavement Care

	Healthcare Professionals (Obstetrician)																			Percentage 7-9
	N	1	%	2	%	3	%	4	%	5	%	6	%	7	%	8	%	9	%	
**Labour and Birth Outcomes**																				
Type of stillbirth	28	1	4%	0	0%	0	0%	0	0%	3	11%	1	14%	4	14%	4	14%	15	54%	82%
Type of birth	28	0	0%	0	0%	0	0%	0	0%	3	11%	0	11%	3	11%	5	18%	17	61%	89%
Complications during birth for mother or baby	28	0	0%	0	0%	0	0%	1	4%	0	0%	3	7%	2	7%	5	18%	17	61%	86%
**Postpartum medical outcomes**																				
Maternal complications after birth	28	0	0%	0	0%	1	4%	1	4%	1	4%	4	11%	3	11%	5	18%	13	46%	75%
Maternal death	28	0	0%	1	4%	1	4%	0	0%	0	0%	1	7%	2	7%	3	11%	20	71%	89%
**Care experience outcomes**																				
Parents' experience of care and support	28	0	0%	0	0%	0	0%	0	0%	1	4%	2	18%	5	18%	7	25%	13	46%	89%
Perceived acknowledgement of parenthood & baby	28	0	0%	0	0%	0	0%	0	0%	2	7%	4	21%	6	21%	5	18%	11	39%	79%
**Investigation outcomes**																				
Uptake of medical investigations performed to understand why a baby died	28	0	0%	0	0%	0	0%	0	0%	1	4%	1	21%	6	21%	4	14%	16	57%	93%
Findings of any medical investigations and cause of death communicated to parents	28	0	0%	0	0%	0	0%	0	0%	1	4%	2	4%	1	4%	6	21%	18	64%	89%
**Grief**																				
Grief	27	0	0%	0	0%	0	0%	0	0%	0	0%	3	7%	2	7%	9	33%	13	48%	89%
**Mental health and Emotional outcomes**																				
Mental Health and Emotional Wellbeing	28	0	0%	0	0%	0	0%	0	0%	0	0%	0	14%	4	14%	8	29%	16	57%	100%
**Whole person outcomes**																				
Quality of life	28	0	0%	0	0%	0	0%	1	4%	0	0%	1	25%	7	25%	5	18%	14	50%	93%

**Social outcomes**																				
Social impact	28	0	%	0	0%	0	0%	0	0%	2	7%	0	32%	9	32%	8	29%	9	32%	93%
Opportunities to talk about stillbirth experience with others	28	0	0%	0	0%	0	0%	0	0%	0	0%	1	29%	8	29%	7	25%	12	43%	96%
Degree of isolation	28	0	0%	0	0%	1	4%	1	4%	0	0%	1	29%	8	29%	8	29%	9	32%	89%
Perceived stigma from community	28	0	0%	0	0%	0	0%	3	11%	1	4%	0	21%	6	21%	8	29%	10	36%	86%
Impact on work	28	0	0%	0	0%	0	0%	2	7%	1	4%	1	18%	5	18%	9	32%	10	36%	86%
**Relationship and support outcomes**																				
Impact on relationship and perceived support from partner and close family	28	0	0%	0	0%	0	0%	0	0%	1	4%	2	11%	3	11%	7	25%	15	54%	89%
**Economic outcomes**																				
Financial costs for parents	27	0	0%	0	0%	0	0%	1	4%	1	4%	2	22%	6	22%	9	33%	8	30%	85%
Financial costs for health service and wider society	26	0	0%	0	0%	0	0%	2	8%	0	0%	2	35%	9	35%	8	31%	5	19%	85%
**Planning subsequent pregnancy outcomes**																				
Perceived support for planning next pregnancy after stillbirth	27	0	0%	0	0%	0	0%	0	0%	1	4%	3	7%	2	7%	5	19%	16	59%	85%

Bereavement Care

	Healthcare Professionals (Nurse Midwives)																			Percentage 7-9
	N	1	%	2	%	3	%	4	%	5	%	6	%	7	%	8	%	9	%	
**Labour and Birth Outcomes**																				
Type of stillbirth	96	1	1%	3	3%	0	0%	2	2%	1	1%	5	5%	6	6%	18	19%	60	63%	88%
Type of birth	93	2	2%	0	0%	0	0%	3	3%	3	3%	8	9%	6	6%	16	17%	55	59%	83%
Complications during birth for mother or baby	94	0	0%	0	0%	0	0%	1	1%	3	3%	3	3%	4	4%	11	12%	72	77%	93%
**Postpartum medical outcomes**																				
Maternal complications after birth	92	2	2%	3	3%	1	1%	1	1%	2	2%	5	5%	3	3%	15	16%	60	65%	85%
Maternal death	91	3	3%	0	0%	1	1%	1	1%	4	4%	3	3%	6	7%	9	10%	64	70%	87%
**Care experience outcomes**																				
Parents' experience of care and support	94	4	4%	0	0%	0	0%	1	1%	4	4%	5	5%	10	11%	17	18%	53	56%	85%
Perceived acknowledgement of parenthood & baby	94	4	4%	2	2%	0	0%	3	3%	4	4%	6	6%	13	14%	22	23%	40	43%	80%
**Investigation outcomes**																				
Uptake of medical investigations performed to understand why a baby died	93	0	0%	0	0%	0	0%	1	1%	4	4%	4	4%	9	10%	12	13%	63	68%	90%
Findings of any medical investigations and cause of death communicated to parents	94	0	0%	1	1%	1	1%	1	1%	1	1%	2	2%	3	3%	19	20%	66	70%	94%
**Grief**																				
Grief	93	2	2%	2	2%	1	1%	1	1%	7	8%	4	4%	4	4%	15	16%	57	61%	82%
**Mental health and Emotional outcomes**																				
Mental Health and Emotional Wellbeing	94	2	2%	2	2%	0	0%	1	1%	1	1%	3	3%	8	9%	20	21%	57	61%	90%
**Whole person outcomes**																				
Quality of life	95	1	1%	1	1%	1	1%	0	0%	2	2%	5	5%	16	17%	26	27%	43	45%	89%
**Social outcomes**																				
Social impact	92	1	1%	1	1%	1	1%	2	2%	5	5%	5	5%	13	14%	24	26%	40	43%	84%
Opportunities to talk about stillbirth experience with others	93	1	1%	1	1%	0	0%	1	1%	3	3%	5	5%	7	8%	25	27%	50	54%	88%
Degree of isolation	90	2	2%	1	1%	1	1%	0	0%	5	6%	4	4%	13	14%	20	22%	44	49%	86%
Perceived stigma from community	93	1	1%	2	2%	1	1%	1	1%	2	2%	7	8%	9	10%	24	26%	46	49%	85%
Impact on work	93	0	0%	3	3%	1	1%	1	1%	1	1%	5	5%	12	13%	25	27%	45	48%	88%

**Relationship and support outcomes**																				
Impact on relationship and perceived support from partner and close family	93	0	0%	1	1%	0	0%	0	0%	2	2%	5	5%	3	3%	21	23%	61	66%	91%
**Economic outcomes**																				
Financial costs for parents	95	1	1%	1	1%	1	1%	2	2%	8	8%	2	2%	13	14%	32	34%	35	37%	84%
Financial costs for health service and wider society	92	1	1%	4	4%	2	2%	5	5%	6	7%	10	11%	11	12%	19	21%	34	37%	70%
**Planning subsequent pregnancy outcomes**																				
Perceived support for planning next pregnancy after stillbirth	92	0	0%	1	1%	0	0%	2	2%	2	2%	5	5%	5	5%	8	9%	69	75%	89%

Bereavement Care

	Parents N	1	%	2	%	3	%	4	%	5	%	6	%	7	%	8	%	9	%	Percentage 7-9
**Labour and Birth Outcomes**																				
Type of stillbirth	53	2	4%	1	2%	0	0%	1	2%	3	6%	2	4%	2	4%	7	13%	35	66%	83%
Type of birth	53	0	0%	2	4%	1	2%	3	6%	1	2%	5	9%	1	2%	6	11%	34	64%	77%
Complications during birth for mother or baby	53	0	0%	0	0%	1	2%	0	0%	3	6%	2	4%	3	6%	5	9%	39	74%	89%
**Postpartum medical outcomes**																				
Maternal complications after birth	53	1	2%	0	0%	0	0%	1	2%	2	4%	2	4%	4	8%	5	9%	38	72%	89%
Maternal death	53	1	2%	0	0%	0	0%	1	2%	3	6%	0	0%	2	4%	8	15%	38	72%	91%
**Care experience outcomes**																				
Parents' experience of care and support	53	1	2%	0	0%	0	0%	1	2%	1	2%	1	2%	3	6%	10	19%	36	68%	92%
Perceived acknowledgement of parenthood & baby	53	3	6%	2	4%	3	6%	2	4%	2	4%	3	6%	5	9%	5	9%	28	53%	72%
**Investigation outcomes**																				
Uptake of medical investigations performed to understand why a baby died	51	1	2%	0	0%	1	2%	1	2%	1	2%	1	2%	1	2%	10	20%	35	69%	90%
Findings of any medical investigations and cause of death communicated to parents	52	0	0%	0	0%	1	2%	2	4%	1	2%	0	0%	4	8%	7	13%	37	71%	92%
**Grief**																				
Grief	53	1	2%	0	0%	1	2%	2	4%	1	2%	2	4%	7	13%	4	8%	35	66%	87%
**Mental health and Emotional outcomes**																				
Mental Health and Emotional Wellbeing	53	1	2%	1	2%	0	0%	0	0%	4	8%	1	2%	5	9%	3	6%	38	72%	87%
**Whole person outcomes**																				
Quality of life	52	1	2%	0	0%	3	6%	0	0%	2	4%	5	10%	5	10%	10	19%	26	50%	79%

**Social outcomes**																				
Social impact	52	2	4%	0	0%	2	4%	2	4%	2	4%	5	10%	8	15%	6	12%	25	48%	75%
Opportunities to talk about stillbirth experience with others	52	0	0%	1	2%	2	4%	1	2%	4	8%	1	2%	4	8%	6	12%	33	63%	83%
Degree of isolation	52	0	0%	1	2%	1	2%	1	2%	2	4%	3	6%	5	10%	8	15%	31	60%	85%
Perceived stigma from community	52	2	4%	0	0%	1	2%	1	2%	1	2%	5	10%	2	4%	7	13%	33	63%	81%
Impact on work	51	2	4%	0	0%	2	4%	0	0%	3	6%	5	10%	3	6%	10	20%	26	51%	76%
**Relationship and support outcomes**																				
Impact on relationship and perceived support from partner and close family	52	0	0%	0	0%	1	2%	2	4%	3	6%	4	8%	5	10%	4	8%	33	63%	81%
**Economic outcomes**																				
Financial costs for parents	51	2	4%	1	2%	1	2%	1	2%	2	4%	5	10%	2	4%	5	10%	32	63%	76%
Financial costs for health service and wider society	51	4	8%	1	2%	1	2%	0	0%	2	4%	8	16%	4	8%	7	14%	24	47%	69%
**Planning subsequent pregnancy outcomes**																				
Perceived support for planning next pregnancy after stillbirth	50	3	6%	0	0%	0	0%	0	0%	1	2%	1	2%	4	8%	7	14%	34	68%	90%

Bereavement Care

	Researchers<br>N	1	%	2	%	3	%	4	%	5	%	6	%	7	%	8	%	9	%	Percentage 7-9
**Labour and Birth Outcomes**																				
Type of stillbirth	39	0	0%	0	0%	1	3%	1	3%	2	5%	3	8%	5	13%	6	15%	21	54%	82%
Type of birth	39	0	0%	0	0%	0	0%	1	3%	3	8%	7	18%	6	15%	6	15%	16	41%	72%
Complications during birth for mother or baby	39	0	0%	0	0%	0	0%	0	0%	1	3%	3	8%	4	10%	11	28%	20	51%	90%
**Postpartum medical outcomes**																				
Maternal complications after birth	40	1	3%	0	0%	1	3%	1	3%	2	5%	5	13%	7	18%	6	15%	17	43%	75%
Maternal death	38	2	5%	2	5%	0	0%	1	3%	1	3%	2	5%	4	11%	7	18%	19	50%	79%
**Care experience outcomes**																				
Parents' experience of care and support	40	0	0%	0	0%	0	0%	0	0%	3	8%	5	13%	4	10%	5	13%	23	58%	80%
Perceived acknowledgement of parenthood & baby	40	0	0%	0	0%	1	3%	0	0%	4	10%	5	13%	8	20%	7	18%	15	38%	75%
**Investigation outcomes**																				
Uptake of medical investigations performed to understand why a baby died	41	0	0%	0	0%	0	0%	2	5%	6	15%	5	12%	2	5%	5	12%	21	51%	68%
Findings of any medical investigations and cause of death communicated to parents	41	0	0%	0	0%	0	0%	0	0%	4	10%	3	7%	5	12%	8	20%	21	51%	83%
**Grief**																				
Grief	40	0	0%	0	0%	0	0%	0	0%	0	0%	5	13%	2	5%	9	23%	24	60%	88%
**Mental health and Emotional outcomes**																				
Mental Health and Emotional Wellbeing	40	0	0%	0	0%	0	0%	0	0%	0	0%	3	8%	4	10%	6	15%	27	68%	93%
**Whole person outcomes**																				
Quality of life	41	0	0%	0	0%	0	0%	0	0%	2	5%	9	22%	2	5%	15	37%	13	32%	73%
**Social outcomes**																				
Social impact	40	0	0%	0	0%	1	3%	0	0%	4	10%	2	5%	9	23%	8	20%	16	40%	83%
Opportunities to talk about stillbirth experience with others	40	0	0%	0	0%	0	0%	0	0%	3	8%	5	13%	7	18%	8	20%	17	43%	80%
Degree of isolation	41	0	0%	0	0%	0	0%	0	0%	2	5%	2	5%	10	24%	9	22%	18	44%	90%
Perceived stigma from community	41	0	0%	0	0%	0	0%	2	5%	1	2%	6	15%	7	17%	8	20%	17	41%	78%
Impact on work	40	0	0%	0	0%	0	0%	1	3%	3	8%	5	13%	7	18%	9	23%	15	38%	78%

**Relationship and support outcomes**																				
Impact on relationship and perceived support from partner and close family	40	0	0%	0	0%	0	0%	0	0%	3	8%	2	5%	5	13%	9	23%	21	53%	88%
**Economic outcomes**																				
Financial costs for parents	40	0	0%	0	0%	2	5%	0	0%	5	13%	4	10%	8	20%	6	15%	15	38%	73%
Financial costs for health service and wider society	39	0	0%	1	3%	0	0%	1	3%	5	13%	4	10%	7	18%	8	21%	13	33%	72%
**Planning subsequent pregnancy outcomes**																				
Perceived support for planning next pregnancy after stillbirth	40	0	0%	0	0%	1	3%	2	5%	3	8%	3	8%	3	8%	10	25%	18	45%	78%
