# Supplementary material for: A meta-core outcome set for stillbirth prevention and bereavement care following stillbirth in LMIC
Source: BMJ Glob Health. 2025 Jan 28;10(1):e017688. doi: 10.1136/bmjgh-2024-017688 (PMC11781104; doi:10.1136/bmjgh-2024-017688)

**Supplementary Figures 1a: Pre-consensus meeting ranking of outcomes: stillbirth prevention (n=16 that completed the exercise).**

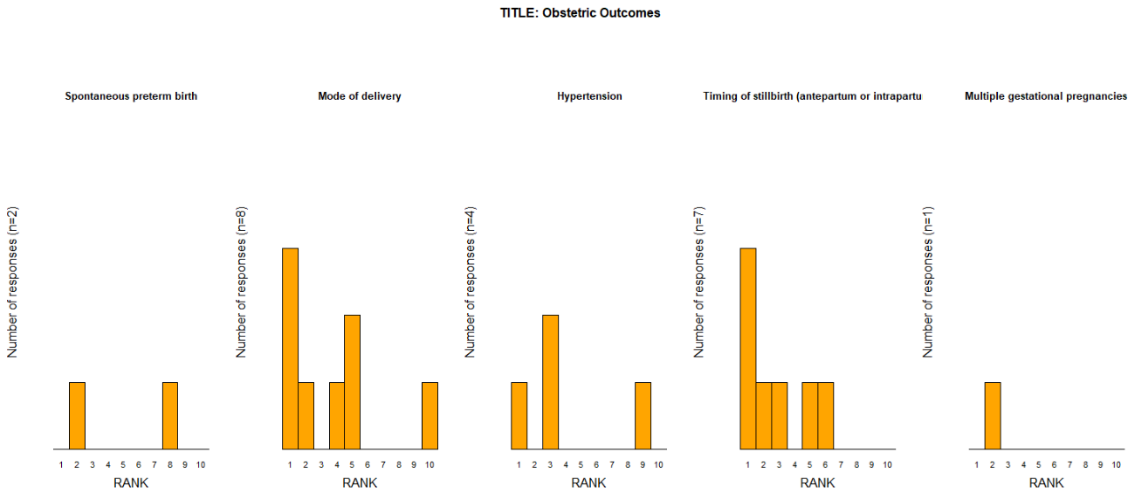


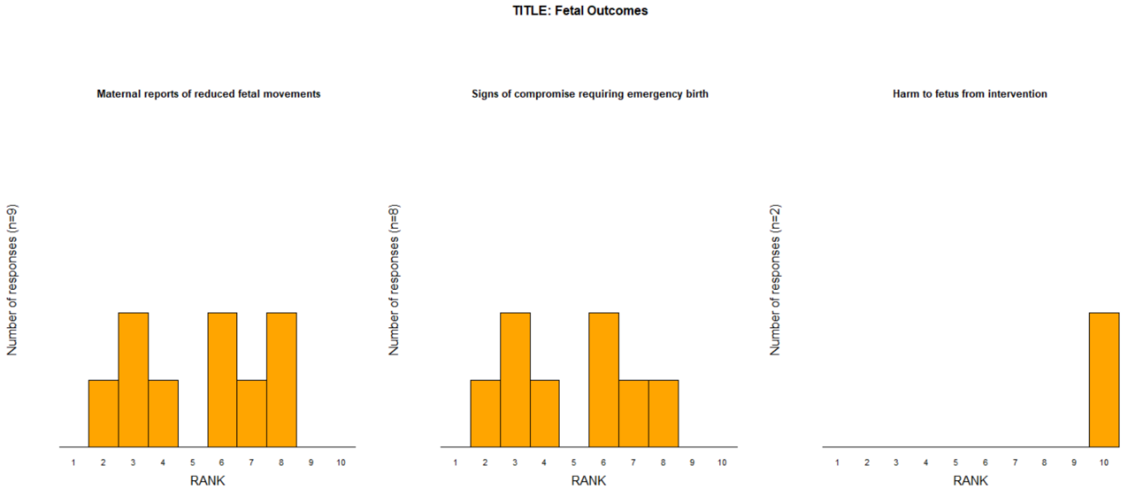

TITLE: Perinatal Outcomes (1)

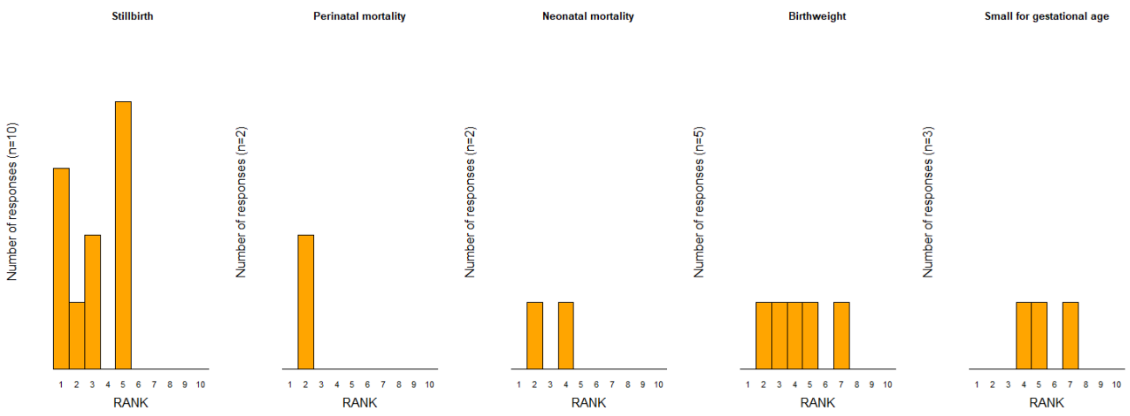


TITLE: Perinatal Outcomes (2)

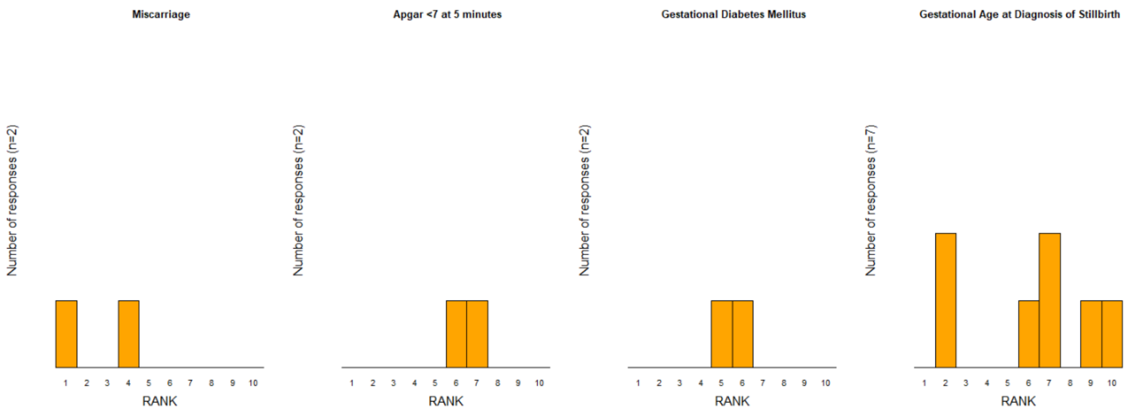


TITLE: Maternal Complications

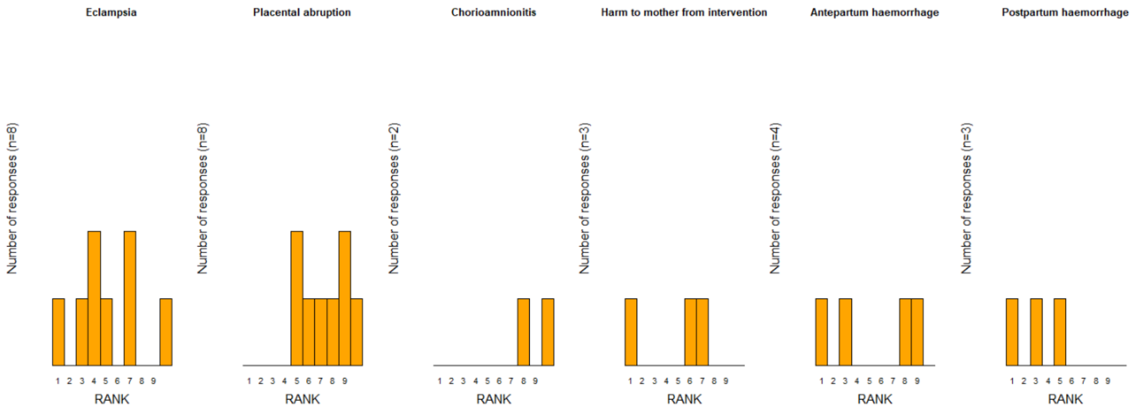

TITLE: Maternal Outcomes

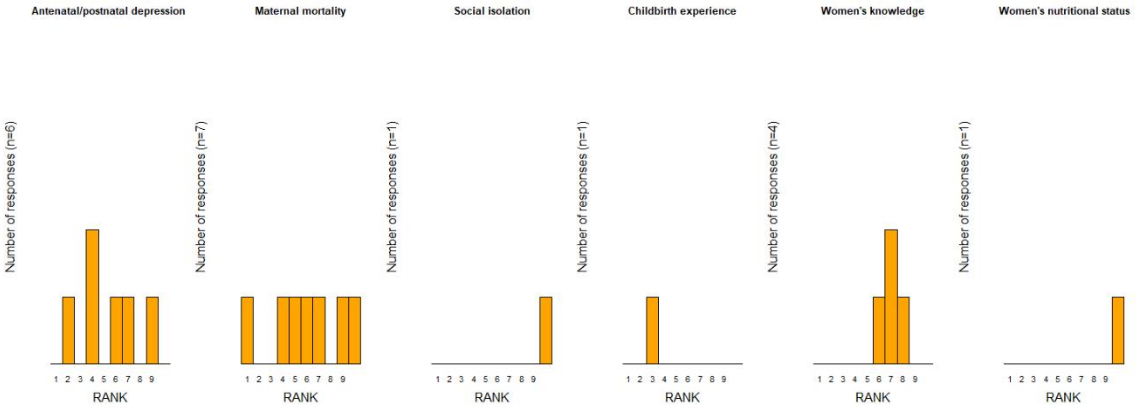


TITLE: Neonatal Complication Outcomes

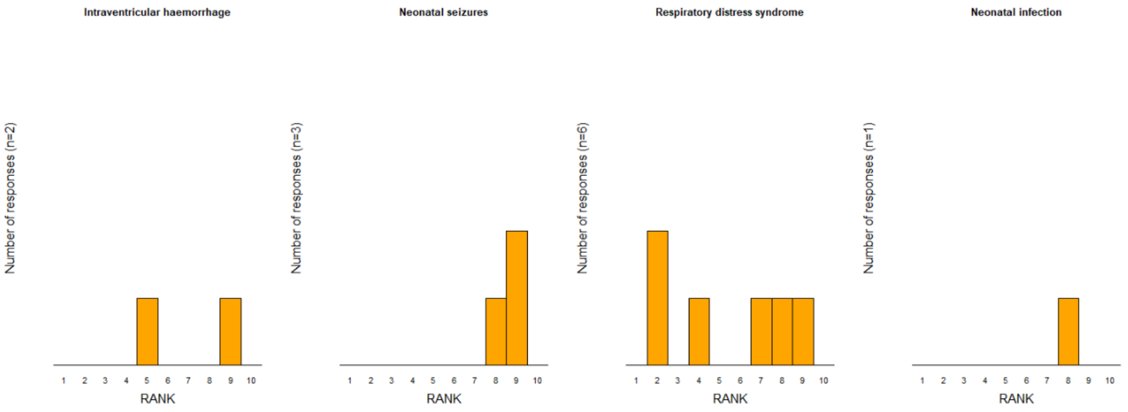

## TITLE: Neonatal Outcomes

Gestational age

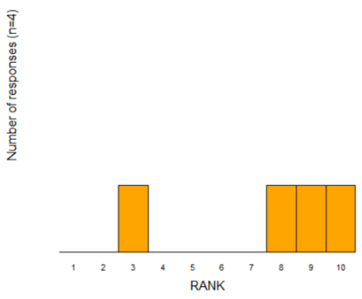


Neonatal intensive care unit (NICU) admission

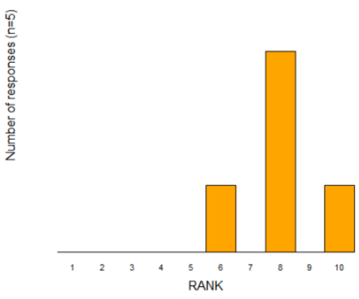


Congenital anomaly

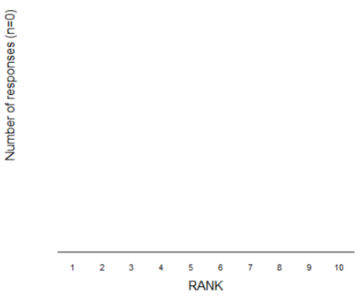


## TITLE: Health Service Outcomes

Number of antenatal visits

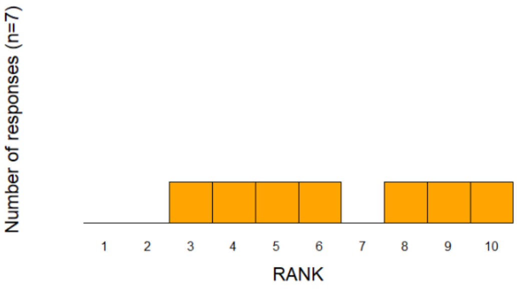


NICU/Special Care Baby Unit length of stay (days)

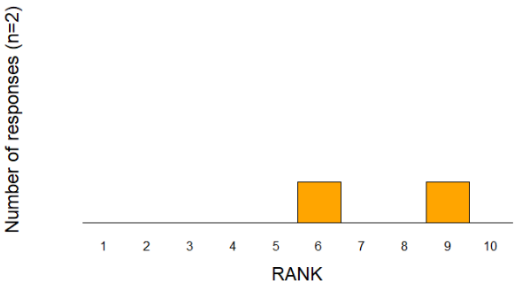


## TITLE: Other

Stigma

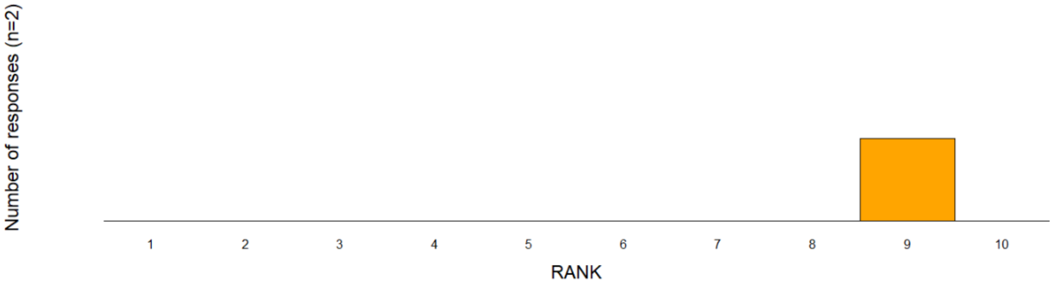

Supplement: online supplemental file 5 [file bmjgh-10-1-s005.pdf]
