# Supplementary material for: A meta-core outcome set for stillbirth prevention and bereavement care following stillbirth in LMIC
Source: BMJ Glob Health. 2025 Jan 28;10(1):e017688. doi: 10.1136/bmjgh-2024-017688 (PMC11781104; doi:10.1136/bmjgh-2024-017688)

Supplementary Figure 1b: Pre-consensus meeting ranking of outcomes: bereavement care following stillbirth (n=15 that completed the exercise).

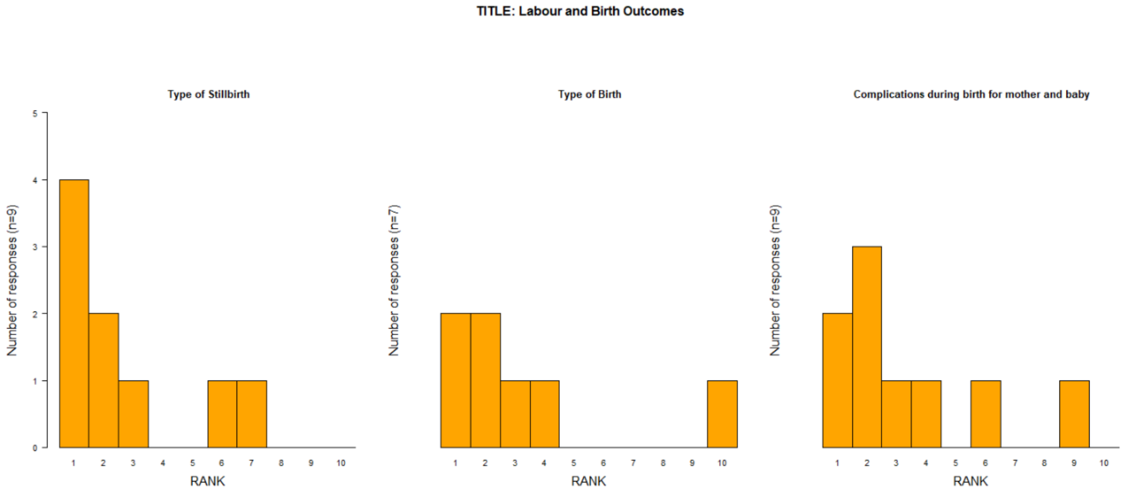

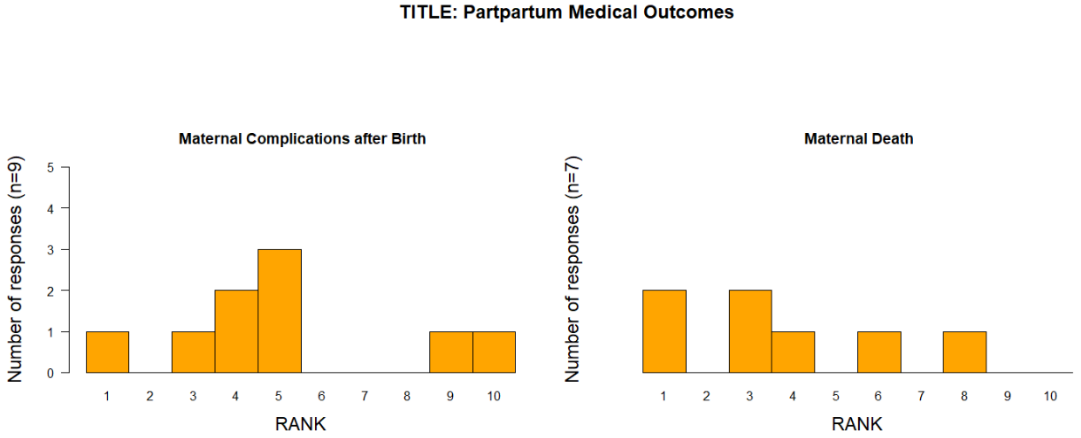

TITLE: Care Experience Outcomes

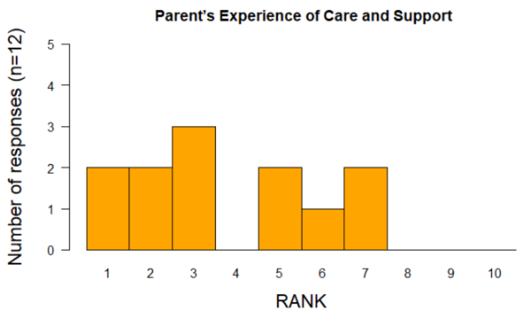

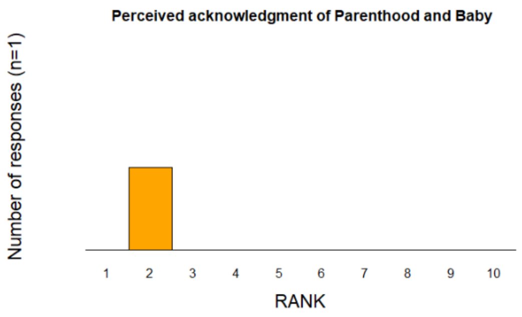

TITLE: Investigation Outcomes

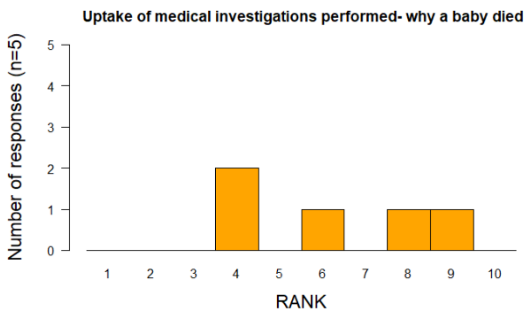

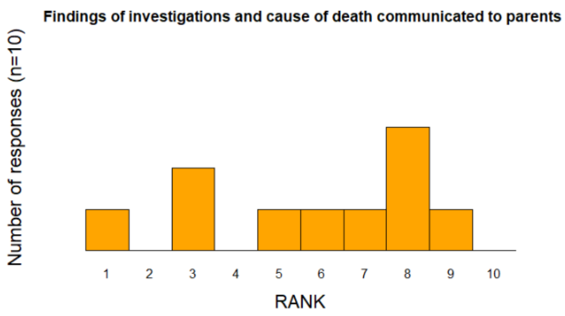

TITLE: Grief

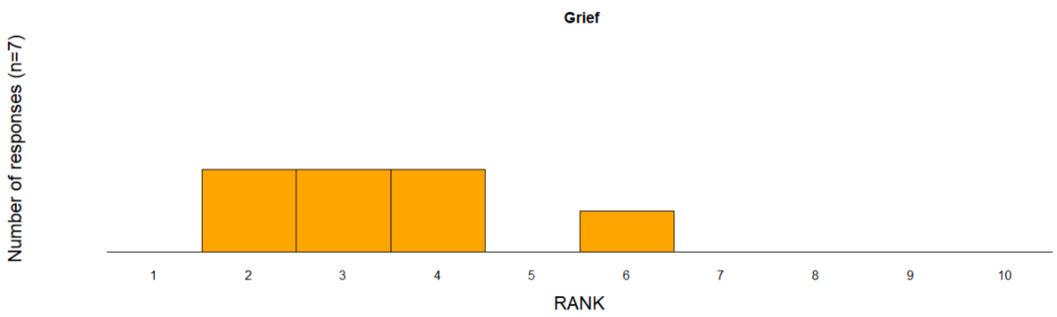

TITLE: Mental Health and Emotional Wellbeing

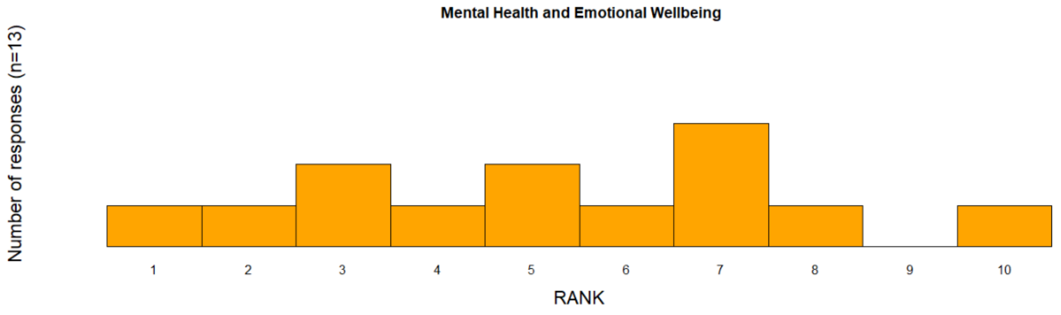

TITLE: Whole Person Outcomes

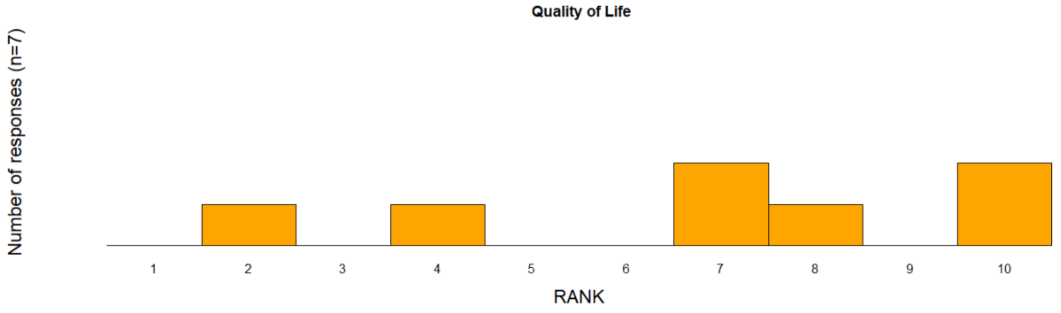

TITLE: Social Outcomes

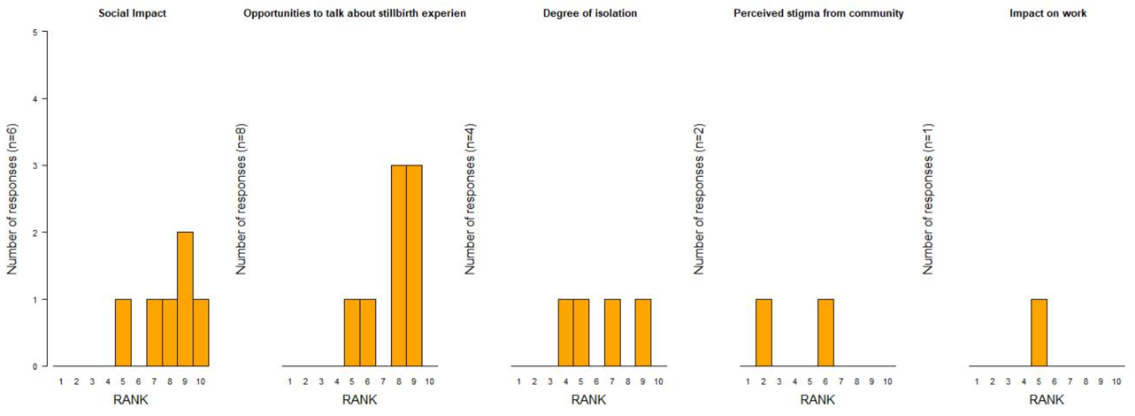

TITLE: Relationship and Support Outcomes

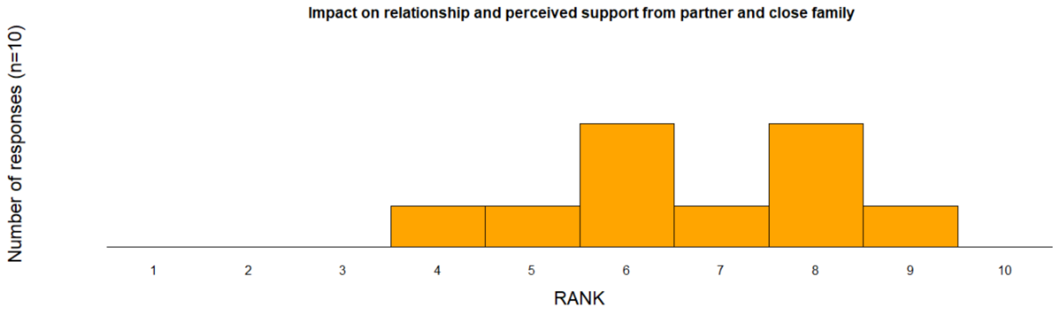

TITLE: Economic Outcomes

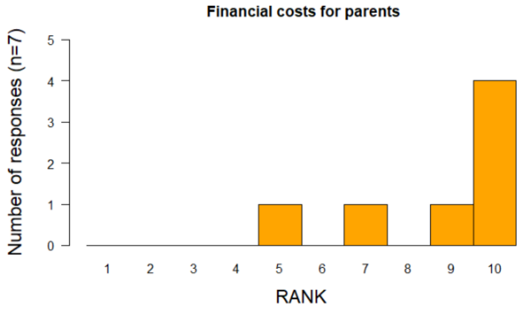

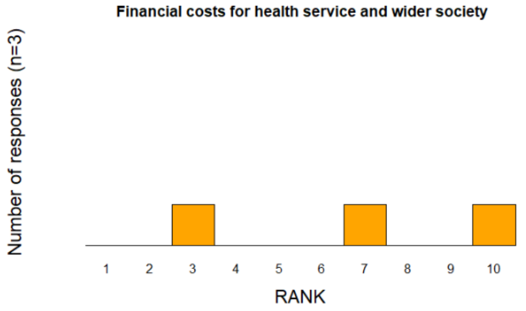

TITLE: Planning subsequent pregnancy outcomes

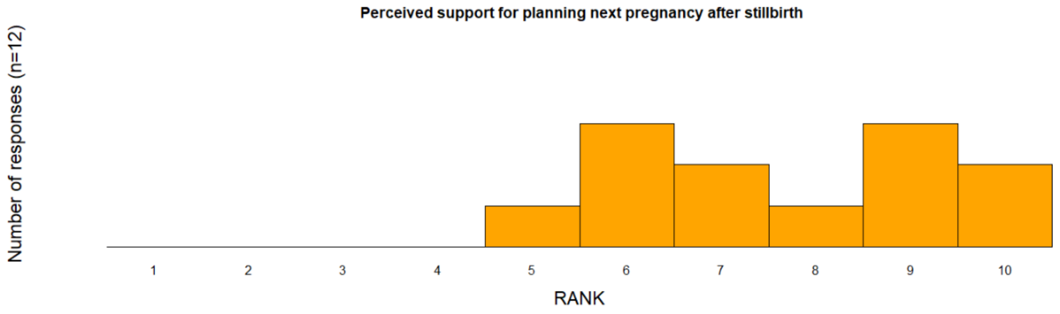

Supplement: online supplemental file 6 [file bmjgh-10-1-s006.pdf]
