# Supplementary material for: A meta-core outcome set for stillbirth prevention and bereavement care following stillbirth in LMIC
Source: BMJ Glob Health. 2025 Jan 28;10(1):e017688. doi: 10.1136/bmjgh-2024-017688 (PMC11781104; doi:10.1136/bmjgh-2024-017688)
Supplement: online supplemental file 7 [file bmjgh-10-1-s007.pdf]

Supplementary Table 3a: Voting decisions and key discussion points during consensus meeting: stillbirth prevention.

80% critical indicates outcome should be in the final core outcome set

Prevention	% critical	Comment	Decision
**Obstetric Outcomes**			
Spontaneous preterm birth	9/11 (82%)	Change to 'Preterm Birth. Following discussion, the majority later voted to exclude based on the inclusion of 'gestational age' in the final COS.	Exclude
Mode of delivery	8/10 (80%)	Change to 'Mode of Birth'.	Include
Hypertension	9/11 (82%)	Following discussion, 100% voted to replace with **pre-eclampsia** as an outcome that is better-defined as a specific disorder that is more likely to be associated with stillbirth.	Include
Timing of stillbirth	10/11 (91%)	Change to '**Type of Stillbirth**' to be recorded as either *antepartum* or *intrapartum.*	Include
Multiple gestational pregnancies	9/12 (75%)	Not supported for inclusion on final reflection.	Exclude
**Fetal Outcomes**			
Maternal reports of reduced foetal movements	10/11 (91%)	Change to '**Reduced foetal movements**'	Include
Signs of compromise requiring emergency birth	10/12 (83%)	Change to '**Emergency birth for foetal compromise**'	Include
Harm to fetus from intervention	7/11 (64%)		Exclude
**Perinatal Outcomes**			
Stillbirth	9/11 (82%)		Include
Perinatal mortality	6/12 (50%)		Exclude
Neonatal mortality	7/12 (58%)	On final reflection of the COS, this outcome was reconsidered as an important *neonatal outcome* and was voted 83% (10/12) critical to include in the COS.	Include
Birthweight	10/12 (83%)		Include
Small for gestational age	8/13 (62%)		Exclude
Miscarriage	8/12 (67%)		Exclude
Apgar <7 at 5 minutes	7/13 (54%)		Exclude
Gestational Diabetes Mellitus	7/12 (58%)		Exclude

Gestational Age at Diagnosis of Stillbirth	10/12 (83%)	Following discussion this was removed as it was felt that this was not needed in the presence of 'Gestational Age at Birth' – see below.	Exclude
**Maternal Complications**			
Eclampsia	10/11 (91%)		Include
Placental abruption	10/11 (91%)		Include
Chorioamnionitis	9/12 (75%)	Outcome largely supported for inclusion in COS but some hesitation that it was too specific. On final reflection it was proposed that '**<u>Severe Maternal Infection</u>**' should be included as an outcome that would absorb Chorioamnionitis. This was supported by more than 80% of the coting participants.	Include
Harm to mother from intervention	7/13 (54%)		Exclude
Antepartum haemorrhage	11/13 (84%)		Include
Postpartum haemorrhage	6/13 (46%)		Exclude
**Maternal Outcomes**			
Antenatal/postnatal depression	7/13 (54%)		Exclude
Maternal mortality	7/12 (58%)		Exclude
Social isolation	4/13 (31%)		Exclude
Childbirth experience	6/13 (46%)		Exclude
Women's knowledge	6/13 (46%)		Exclude
Women's nutritional status	8/14 (57%)		Exclude
**Neonatal Complication**			
Intraventricular haemorrhage	7/13 (54%)		Exclude
Neonatal seizures	8/13 (62%)		Exclude
Respiratory distress syndrome	9/13 (69%)		Exclude
Neonatal infection	7/13 (54%)		Exclude
**Neonatal Outcome**			
Gestational age	11/14 (79%)	Change to '**<u>Gestational Age at Birth</u>**'. In a research setting, this may help determine if any intervention may have impacted on a stillbirth outcome.	Include
Neonatal intensive care unit (NICU) admission	9/13 (69%)		Exclude
Congenital anomaly	5/13 (38%)		Exclude
**Health Service Outcome**			

Number of antenatal visits	4/12 (33%)	Exclude
NICU/Special Care Baby Unit length of stay (days)	4/12 (33%)	Exclude
**Other**		
Stigma	2/12 (17%)	Exclude
