# Supplementary material for: A meta-core outcome set for stillbirth prevention and bereavement care following stillbirth in LMIC
Source: BMJ Glob Health. 2025 Jan 28;10(1):e017688. doi: 10.1136/bmjgh-2024-017688 (PMC11781104; doi:10.1136/bmjgh-2024-017688)
Supplement: online supplemental file 8 [file bmjgh-10-1-s008.pdf]

Supplementary Table 3b: Voting decisions and key discussion points during consensus meeting: bereavement care following stillbirth.

80% critical indicates outcome should be in the final core outcome set

Prevention	% critical	Comment	Decision
**Labour and Birth Outcomes**			
Type of stillbirth	8/10 (80%)		Include
Type of birth	5/10 (50%)		Exclude
Complications during birth for mother or baby	10/11 (91%)	Panel members felt this outcome was important as it can help answer or explain the reason for stillbirth. After further discussions it was felt the core outcome should be '**Obstetric Fistula**' as this is the main evidence-based complication associated with stillbirth – this was voted as 100% (10/10) in favour. Physical injury to the baby was also raised as a singular important complication but the vote did not reach critical (64%).	Include
**Postpartum Medical Outcomes**			
Maternal complications after birth	5/12 (42%)		Exclude
Maternal death	4/11 (36%)		Exclude
**Care Experience Outcomes**			
Parents' experience of care and support	9/11 (82%)		Include
Perceived acknowledgement of parenthood & baby	2/11 (27%)		Exclude
**Investigation Outcomes**			
Uptake of medical investigations performed to understand why a baby died	9/13 (69%)	Important to understand the cause of death but many investigations not available in all LMIC settings.	Exclude
Findings of any medical investigations and cause of death communicated to parents	8/12 (67%)		Exclude
**Grief**			
Grief	7/10 (70%)	Arguments for and against inclusion, but ultimately this outcome did not meet the inclusion criteria. Potentially the first emotion experienced that can lead to potentially more important outcomes downstream, e.g. mental health.	Exclude
**Mental Health and Emotional Outcomes**			

Mental Health and Emotional Wellbeing	6/11 (55%)	It was felt this outcome was critical as you cannot provide bereavement care without recording mental health and emotional wellbeing. It was felt that the initial definition was too broad and should not include drugs and alcohol use in an LMIC setting. On a revote excluding these aspects, 92% (11/12) voted the outcome into the core outcome set.	Include
**Whole person outcomes**			
Quality of life	5/15 (33%)		Exclude
**Social outcomes**			
Social impact	3/12 (25%)		Exclude
Opportunities to talk about stillbirth experience with others	4/10 (40%)		Exclude
Degree of isolation	5/12 (42%)		Exclude
Perceived stigma from community	3/12 (25%)		Exclude
Impact on work	5/14 (36%)		Exclude
Social wellbeing	10/11 (91%)	On final reflection of the agreed COS, it was felt that something around social care was missing but the social outcomes voted out were perhaps not defined well enough and too specific. A new outcome was proposed as '**Social Wellbeing**', defined and voted in a critical to include.	Include
**Relationship and support outcomes**			
Impact on relationship and perceived support from partner and close family	6/14 (43%)		Exclude
**Economic outcomes**			
Financial costs for parents	6/13 (46%)		Exclude
Financial costs for health service and wider society	2/14 (14%)		Exclude
**Planning subsequent pregnancy outcomes**			
Perceived support for planning next pregnancy after stillbirth	8/13 (62%)		Exclude
